# Recent Advances in Lipase-Mediated Preparation of Pharmaceuticals and Their Intermediates

**DOI:** 10.3390/ijms161226191

**Published:** 2015-12-11

**Authors:** Ana Caroline Lustosa de Melo Carvalho, Thiago de Sousa Fonseca, Marcos Carlos de Mattos, Maria da Conceição Ferreira de Oliveira, Telma Leda Gomes de Lemos, Francesco Molinari, Diego Romano, Immacolata Serra

**Affiliations:** 1 Laboratório de Biotecnologia e Síntese Orgânica (LABS), Department of Organic and Inorganic Chemistry, Federal University of Ceará, Campus do Pici, Postal box 6044, 60455-970 Fortaleza, Ceará, Brazil; carol.luz@hotmail.com (A.C.L.M.C.); thiagofonseka@hotmail.com (T.S.F.); mcfo@ufc.br (M.C.F.O.); tlemos@dqoi.ufc.br (T.L.G.L.); 2 Department of Food, Environmental and Nutritional Sciences (DEFENS), University of Milan, via Mangiagalli 25, 20133 Milan, Italy; francesco.molinari@unimi.it (F.M.); diego.romano@unimi.it (D.R.); Immacolata.serra@unimi.it (I.S.)

**Keywords:** biocatalysis, lipases, kinetic resolution, enantioselectivity, pharmaceuticals

## Abstract

Biocatalysis offers an alternative approach to conventional chemical processes for the production of single-isomer chiral drugs. Lipases are one of the most used enzymes in the synthesis of enantiomerically pure intermediates. The use of this type of enzyme is mainly due to the characteristics of their regio-, chemo- and enantioselectivity in the resolution process of racemates, without the use of cofactors. Moreover, this class of enzymes has generally excellent stability in the presence of organic solvents, facilitating the solubility of the organic substrate to be modified. Further improvements and new applications have been achieved in the syntheses of biologically active compounds catalyzed by lipases. This review critically reports and discusses examples from recent literature (2007 to mid-2015), concerning the synthesis of enantiomerically pure active pharmaceutical ingredients (APIs) and their intermediates in which the key step involves the action of a lipase.

## 1. Introduction

Among the applications of lipases, the synthesis of enantiomerically pure active pharmaceutical ingredients (APIs) and their intermediates using these enzymes is a subject of continuing interest. It is noteworthy that most of the drugs are chiral, and it is important to know which stereoisomer has the desired biological activity. Thus, only the active stereoisomer is administered, thereby preventing the patient receiving a dose of stereoisomer with unnecessary activity. Such practice avoids unnecessary consumption of the substance and minimizes side effects. Biocatalysis offers an alternative approach to conventional chemical processes for the production of enantiomerically pure chiral drugs. Lipases are highlighted as being among the most frequently used enzymes for the production of drugs and their intermediates, a subject covered in an elegant review in 2006 [1]. The use of lipases is mainly due to the characteristics of the regio-, chemo- and enantioselectivity in the resolution process of racemates, without the use of cofactors. Moreover, this class of enzymes has generally excellent stability in the presence of organic solvents, facilitating the solubility of the organic substrate to be modified [1]. Some reviews on the preparation of APIs via biocatalysis were also published, but involved various types of enzymes and not only lipases [2,3,4,5,6].

On this topic, we will present representative examples of recent reports in the literature (2007 to mid-2015) concerning the lipase-catalyzed preparation of APIs and their intermediates through hydrolytic (Section 2) and esterification (Section 3) approaches. Additionally, examples involving both approaches as complementary methods are discussed (Section 4).

## 2. Hydrolytic Approach

Hydrolysis of racemic or prochiral esters catalyzed by lipases for preparing an enantiomerically pure drug intermediate is a well-established method in organic chemistry; nevertheless, new applications, new enzymes, and improved techniques for bettering the efficiency of already known lipase-catalyzed hydrolysis are continuously proposed.

### 2.1. Key Intermediates of Paclitaxel Side Chain

**Scheme 1:**
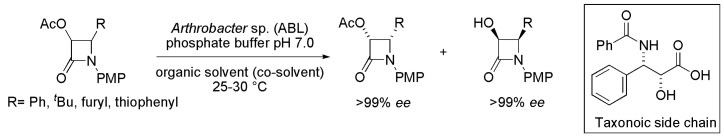
Kinetic enzymatic hydrolysis of racemic arylazetidiones, precursors of amino acid side chain of Paclitaxel [7].

An interesting example of the application of lipases for preparing a series of useful chiral intermediates was proposed for the chemoenzymatic synthesis of analogues of Paclitaxel (trade names: Taxol or Onxal). This compound is a polyoxygenated diterpenoid isolated from the bark of *Taxus brevifolia*, which has been extensively used in anti-cancer therapy. A number of compounds from the class of arylazetidiones, precursors of the amino acid side chain of Paclitaxel, as well as a new generation of taxanoides side chains, were prepared in enantiomerically pure form by kinetic resolution, via hydrolysis, of the corresponding esters in the presence of lipases. A screening was performed with 14 lipases, and four of them (PS (*Burkholderia cepacia*), PS-C (*B. cepacia* immobilized on ceramics), AS (*Aspergillus niger*), and ABL (*Arthrobacter* sp.)) showed positive results. Among these lipases, *Arthrobacter* sp. (ABL) was the only one that hydrolyzed all tested substrates. ABL was used on the resolution of four arylazetidiones derivatives (Scheme 1) in buffer solution pH 7.0, in the presence of co-solvents such as acetonitrile (MeCN), dimethylformamide (DMF) or dimethylsulfoxide (DMSO), and resulted in enantiomeric excess of both alcohols and acetates of >99% and conversions close to 50% [7].

**Scheme 2:**
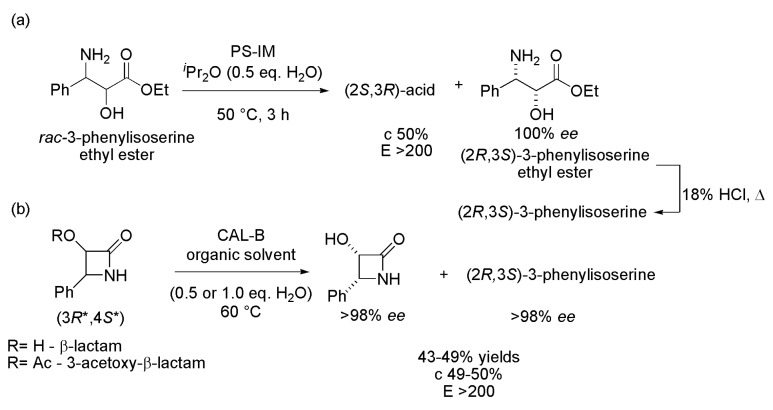
Synthesis of (2*R*,3*S*)-3-phenylisoserine, key intermediates of Paclitaxel side chain, via enzymatic hydrolysis of the (**a**) racemic ethyl 3-amino-3-phenyl-2-hydroxypropionate [7] or (**b**) β-lactams [10].

The compound (2*R*,3*S*)-3-phenylisoserine is also a key intermediate of the Paclitaxel side chain, and it has been already produced by biocatalytical methods [8]. The racemate of ethyl 3-amino-3-phenyl-2-hydroxy-propionate (3-phenylisoserine ethyl ester) was synthesized and resolved via lipase-mediated kinetic hydrolysis. Various lipases were screened and the best result (c 50%, *E* > 200) was obtained with lipase from *B. cepacia* immobilized on diatomaceous earth (PS IM). After optimizing the reaction conditions (diisopropyl ether (DIPE) with 0.5 eq. H_2_O as solvent, 50 °C), 3 h, the (2*R*,3*S*)-3-amino-3-phenyl-2-hydroxy-propionate was obtained with 100% *ee*, c 50% and *E* > 200. Subsequent hydrolysis of this latter compound led to (2*R*,3*S*)-3-phenylisoserine (Scheme 2a). One advantage of this strategy is that it was not necessary to protect the amino group [9].

Another strategy to produce the (2*R*,3*S*)-3-phenylisoserine involved the enantioselective ring-cleavage of (3R*,4S*)-β-lactam (R=H) and its 3-acetoxy derivative (R=Ac) using CAL-B (lipase B from *Candida antarctica* produced by the submerged fermentation of a genetically modified *Aspergillus oryzae* and absorbed on a macroporous resin) as an enzyme at 60 °C (Scheme 2b). The kinetic hydrolyses were performed on gram-scale (1.0 g of substrate) after optimizing the reaction conditions for each starting material. The enantioselective ring-cleavage of the (3R*,4S*)-β-lactams (R=H) in *tert*-butylmethyl ether (TBME) (with 0.5 eq. of water) yielded, after 18 h, the (3*S*,4*R*)-β-lactam (>99% *ee*, 48% yield) and (2*R*,3*S*)-3-phenylisoserine (98% *ee*, 48% yield). These two compounds were also formed with high enantioselectivity (>98% *ee*) and good yields (43% and 49%, respectively) when the reaction was performed with the (3R*,4S*)-3-acetoxy-β-lactam (R=Ac). In this case, the solvent was DIPE (1.0 eq. H_2_O) and, after 50 h, the starting material was 100% converted into the enantiopure products (>98% *ee*) [10].

### 2.2. Key Intermediate of Crizotinib

**Scheme 3:**
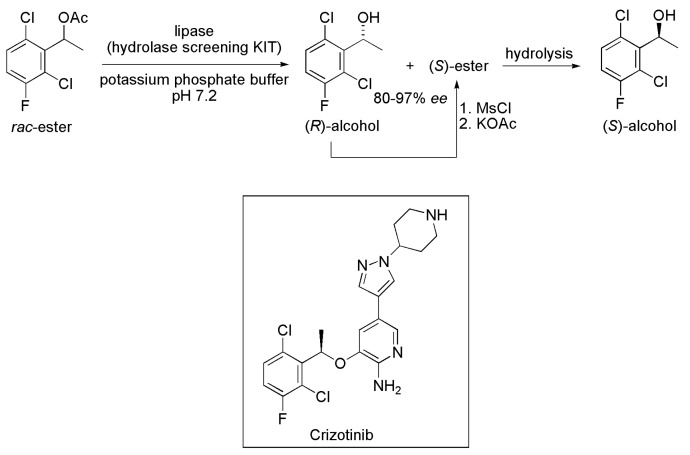
Synthesis of (*S*)-1-(2,6-dichloro-3 fluorophenyl)ethanol via kinetic enzymatic hydrolysis of the corresponding racemic ester [12].

An extensive screening among commercial lipases and esterases was carried out by researchers at Agouron Pharmaceuticals for the preparation of (*S*)-1-(2,6-dichloro-3-fluorophenyl)ethanol from its corresponding racemic ester (Scheme 3); this chiral intermediate can be linked with an ether bond to different 2-aminopyridine compounds that potently inhibit auto-phosphorylation of human heptocyte growth factor receptor. In addition, it is an intermediate on the preparation of the potent antitumor compound Crizotinib [11]. Highly enantioselective hydrolysis (*E* > 100) was observed with different commercial lipases (CAL-B and *Rhizopus delemar* lipase, among the others), and provided the (*S*)-ester and (*R*)-alcohol with *ee* ranging from 80% to 97%. Then, these two compounds were not separated, and the mixture was subjected to mesylation reaction (conversion of the (*R*)-alcohol into its mesyl derivative), followed by treatment with potassium acetate to exclusively yield the (*S*)-ester. Hydrolysis of this compound furnished the desired product (*S*)-1-(2,6-dichloro-3-fluorophenyl)ethanol in high yield [12].

### 2.3. Pregabalin and Analogues

**Scheme 4:**
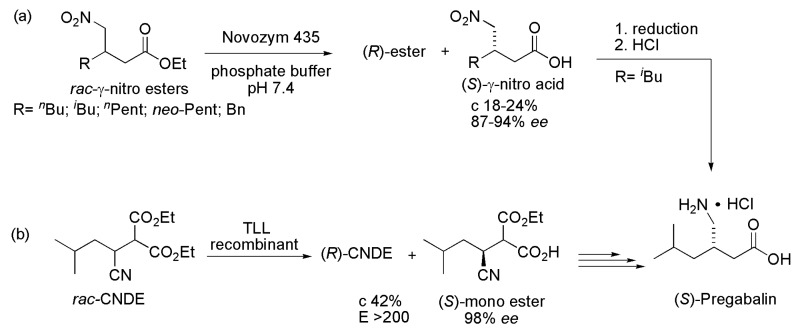
Synthesis of Pregabalin intermediates through kinetic enzymatic hydrolysis of (**a**) *rac*-γ-nitro esters [13] and (**b**) *rac*-2-carboxyethyl-3-cyano-5-methylhexanoic acid ethyl ester [14].

Pregabalin ((*S*)-3-(aminomethyl)-5-methylhexanoic acid, trade name Lyrica^®^) is a drug used in anticonvulsant therapy. Pregabalin and its analogues were obtained by hydrolysis of γ-nitro esters, followed by hydrogenation of the nitro group on Raney nickel. Hydrolysis of the γ-nitro esters can be effectively catalyzed by lipase Novozym 435, as seen in Scheme 4a, affording (*S*)-γ-nitro acids with moderate conversion (18%–24%) and *ee* ranging between 87% and 94% [13]. Additionally, 2-carboxyethyl-3-cyano-5-methylhexanoic acid ethyl ester (CNDE) was enantioselectively hydrolyzed as the key step in the preparation of Pregabalin (Scheme 4b). This biotransformation was achieved by using *Thermomyces lanuginosus* lipase (TLL), the enzyme contained in commercial Lipolase® and Lipozyme TL IM^®^. Recombinant lipase from *T. lanuginosus* DSM 10635 showed excellent (*S*)-enantioselectivity to CNDE (*E* > 200), but with low catalytic activity. The enzyme was evolved by protein engineering, allowing the preparation of (3*S*)-2-carboxyethyl-3-cyano-5-methylhexanoic acid with 42% conversion and 98% *ee*, starting from 255 g/L of substrate [14].

**Scheme 5:**
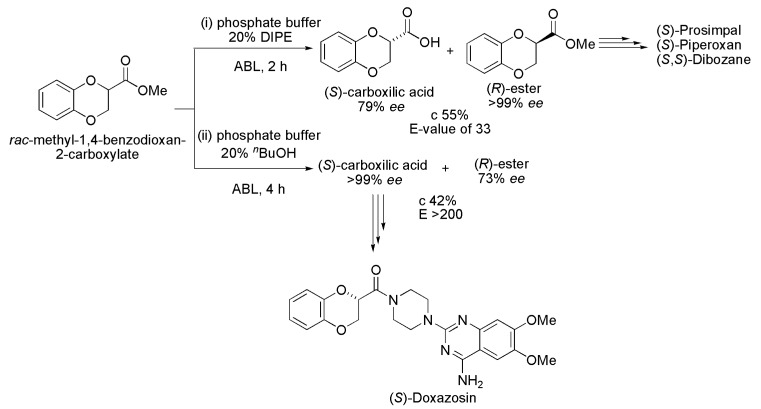
Kinetic enzymatic hydrolysis of racemic methyl-1,4-benzodioxan-2-carboxylate, affording intermediates used in the synthesis of (*S*)-Prosimpal, (*S*)-Piperoxan, (*S*,*S*)-Dibozane and (*S*)-Doxazosin [15].

### 2.4. Prosimpal, Piperoxan, Dibozane and Doxazosin

The drugs (*S*)-Prosimpal, (*S*)-Piperoxan and (*S*,*S*)-Dibozane are α-adrenergic receptor antagonists, and (*S*)-Doxazosin is a drug used in the treatment of hypertension. All (*S*)-isomers are more effective than the corresponding (*R*)-isomers. The aforementioned drugs were synthesized by hydrolytic kinetic resolution of the racemic intermediate methyl-1,4-benzodioxan-2-carboxylate in the presence of lipase from whole cells of wild species of *Arthrobacter* (ABL), as seen in Scheme 5. The reactions were performed in two different conditions: (i) 0.1 M phosphate buffer with 20% DIPE as a co-solvent and (ii) 0.1 M phosphate buffer with 20% of *n*-butanol as a co-solvent. In the first reaction condition, after 2 h of reaction, the corresponding (*S*)-carboxylic acid (79% *ee*) and the (*R*)-methyl ester (>99% *ee*) were obtained with c 55% and *E*-value of 33. In the second reaction condition, after 4 h, (*S*)-carboxylic acid (>99% *ee*) and (*R*)-methyl ester (73% *ee*) were obtained with c 42% and *E* > 200. The syntheses of the pharmaceuticals (*S*)-Prosimpal, (*S*)-Piperoxan and (*S*,*S*)-Dibozane were performed by using the (*R*)-methyl ester (>99% *ee*) obtained from the first reaction condition, while the (*S*)-carboxylic acid (>99% *ee*) obtained in the second condition was used in the synthesis of (*S*)-Doxazosin [15].

### 2.5. Key Intermediate of Ezetimibe

**Scheme 6:**
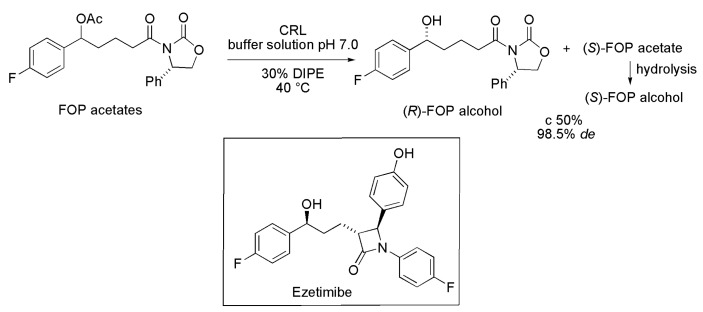
Kinetic enzymatic hydrolysis of diastereoisomeric mixture of FOP acetates to produce the (*S*)-FOP alcohol used in the synthesis of the drug Ezetimibe [16].

Ezetimibe is a drug used in the reduction of cholesterol and blood lipids. The synthesis of this drug requires the enantiopure 3-[5-(4-fluorophenyl)-5(*S*)-hydroxypentanoyl]-4(*S*)-4-phenyl-1,3-oxazolidin-2-one ((*S*)-FOP alcohol) as a key intermediate. Kinetic resolution of the diastereoisomeric mixture of FOP acetates was assessed using several commercial lipases; the most efficient lipase was from *Candida rugosa* (CRL) and the best reaction conditions were buffer solution (pH 7.0) containing 30% of DIPE as a co-solvent, at 40 °C, and an enzyme:substrate (*w*/*w*) ratio of 2.5:1. In such conditions, after reaching 50% conversion, it was possible to obtain (*S*)-FOP acetate with diastereomeric excess (*de*) 98.5% (Scheme 6). The same results were obtained when the method was applied to scale-up, starting from 1 g of the diastereoisomeric mixture of FOP acetates [16].

### 2.6. Key Intermediate of Hepatitis C Virus Protease Inhibitor

**Scheme 7:**
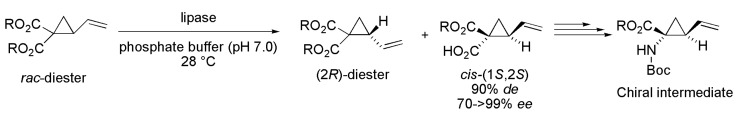
Kinetic enzymatic hydrolysis of dialkyl 2-vinyl-1,1-cyclopropanedicarboxylate to produce the *cis*-(1*S*,2*S*)-1-alcoxycarbonyl-2-vinylcyclopropane-1-carboxylic acid used to obtain the key intermediate in the synthesis of the virus protease inhibitors of Hepatitis C [17].

The chiral compound *trans*-alkyl (1*R*,2*S*)-1-(*tert*-butoxycarbonylamino)-2-vinylcyclopropanecarboxylate is an important intermediate for the synthesis of certain Hepatitis C virus protease inhibitors. Initially, *rac*-dialkyl 2-vinyl-1,1-cyclopropanedicarboxylate was submitted to a kinetic resolution in the presence of various hydrolytic enzymes including lipases (lipase OF from *Candida rugosa*; lipase from *Mucor miehei*; lipase OF from *C. rugosa* (formerly *C. cylindracea*); and lipase N from *Rhizopus niveus*). The reactions were performed in phosphate buffer, pH 7.0, at 28 °C. As results, the remaining dialkyl (2*R*)-2-vinylcyclopropane-1,1-dicarboxylate and the hydrolysis product, the 1-alcoxycarbonyl-2-vinylcyclopropane-1-carboxylic acid, were obtained with diastereomeric excess values (*de*) >90% in favor of the *cis*-isomer (ester/vinyl group), and enantiomeric excess values (*ee*) ranging from 70% to >99% in favor of the *cis*-(1*S*,2*S*) enantiomer (Scheme 7). After a few chemical steps, it was possible to obtain the key chiral intermediate for the synthesis of the virus protease inhibitors of Hepatitis C, *trans*-alkyl (1*R*,2*S*)-1-(*tert*-butoxycarbonylamino)-2-vinylcyclopropanecarboxylate [17].

### 2.7. Naproxen

In the last few years, a number of examples have been reported concerning the application of immobilized lipases for achieving highly efficient stereoselective hydrolysis of esters of pharmaceutical interest.

**Scheme 8:**
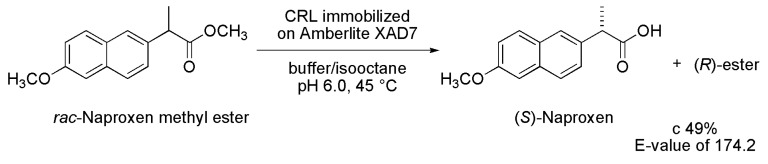
Synthesis of (*S*)-Naproxen through kinetic enzymatic hydrolysis of the racemic Naproxen methyl ester [18].

A classic example of the use of immobilized lipase in hydrolysis is the obtainment of (*S*)-Naproxen ((*S*)-(+)-2-(6-methoxy-2-naphthyl) propionic acid) from racemic Naproxen methyl ester. (*S*)-Naproxen belongs to a class of non-steroidal anti-inflammatory drugs and their activity is 28-fold higher than the corresponding (*R*)-enantiomer. This drug was prepared from enzymatic hydrolysis of racemic Naproxen methyl ester. The kinetic resolution was carried out in the presence of lipase from *Candida rugosa* immobilized on Amberlite XAD7 (CRL type VII). The best results were obtained at 45 °C in an aqueous phase/isooctane biphasic batch system, at pH 6.0, a lipase load of 800 U/mL and a substrate concentration of 10 mg/mL. Under these conditions, a conversion of 49% and an *E*-value of 174.2 were reached (Scheme 8) [18].

The same biotransformation was improved by using nanoparticles as additives for the encapsulation of the enzyme via the sol–gel method. The encapsulated lipase showed outstanding enantioselectivity, with an *E*-value ranging from 265 to 371 and >98% *ee* depending on the sol–gel encapsulation process employed [19].

### 2.8. Key Intermediate of Prostaglandins, Prostacyclins and Thromboxane

**Scheme 9:**
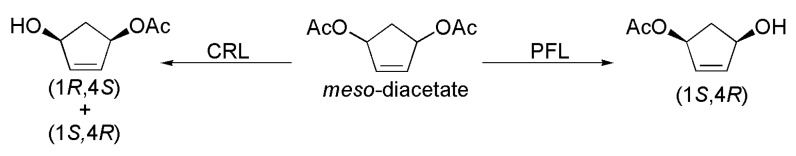
Desymmetrization of *meso*-cyclopent-2-en-1,4-diacetate by enzymatic hydrolysis [20].

The compound (1*S*,4*R*)-4-hydroxycyclopent-2-enyl acetate is an important intermediate in the synthesis of cyclopentenoid molecules with important biological activity, such as prostaglandins, prostacyclins and thromboxane. Enzymatic hydrolysis of *meso*-cyclopent-2-en-1,4-diacetate may give access to (1*S*,4*R*)-4-hydroxycyclopent-2-enyl acetate, taking advantage of the so-called *meso*-trick. Lipases from *Pseudomonas fluorescens* (PFL) and *Candida rugosa* (CRL) were immobilized by physical adsorption or by chemical functionalization on core-shell superparamagnetic nanoparticles, and their performances were compared with the ones of the free enzymes. The biotransformations were performed in a two-liquid-phase system composed with 80% hexane and 20% water under mild end-over-end rotation. CRL was poorly enantioselective, while free and immobilized PFL afforded enantiopure (1*S*,4*R*)-4-hydroxycyclopent-2-enyl acetate (Scheme 9). The re-use of the differently nanoparticle-immobilized PFL showed that the activity of physically adsorbed PFL decreased more than 50% after two cycles, whereas the chemically immobilized enzyme was much more stable [20].

### 2.9. Ketoprofen

**Scheme 10:**
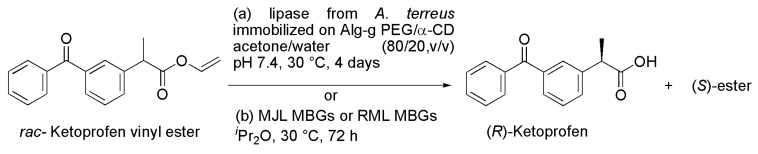
Kinetic enzymatic hydrolysis of racemic Ketoprofen vinyl ester, a member of the class of nonsteroidal anti-inflammatory drugs (NSAIDs), using lipases immobilized on (**a**) Alg-*g*-PEG/α-CD [21] or in (**b**) micro-emulsion-based organogels (MBGs) [22].

Only the (*S*)-enantiomer of Ketoprofen (2-(3-benzoylphenyl)propionic acid) is therapeutically relevant as a nonsteroidal anti-inflammatory drug (NSAID). Kinetic resolution of racemic Ketoprofen vinyl ester was obtained by enzymatic hydrolysis using a lipase from *Aspergillus terreus* immobilized on modified alginate and cyclodextrin hollow spheres [21]. Under optimized conditions (enzyme immobilized on Alg-*g*-PEG/α-CD hollow spheres used in acetone/water 80/20, pH 7.4, at 30 °C), the biotransformation was enantioselective (*E*-value of 129), furnishing (*R*)-Ketoprofen (96% *ee* at 46% conversion). Under the same conditions, free *A. terreus* lipase gave only 16% conversion with low enantioselectivity (*E*-value of 11.4), as seen in Scheme 10a and Table 1. Notably, immobilized *A. terreus* lipase was continuously used up to 20 cycles with minimal loss of activity, whereas the free enzyme cannot be recycled.

A new approach to resolve racemic Ketoprofen vinyl ester was developed after comparison of the performances of different lipases (from *Mucor javanicus*, *Rhizomucor miehei*, *Candida rugosa* and *Pseudomonas cepacia*) employed both as free enzymes or immobilized in micro-emulsion-based organogels (MBGs) [22]. The reactions were conducted in the presence of DIPE at 30 °C. The bioconversion with free lipases generally provided low conversions (maximum 12%) compared with the immobilized lipases (maximum yield 49%). Immobilized *Mucor javanicus* lipase (MJL) exhibited the highest enantioselectivity (*E* > 200), whereas immobilized *Rhizomucor miehei* lipase (RML) displayed a moderate *E*-value (35), but proved to be tolerant to various organic solvents such as DIPE, TBME, tetrahydrofuran (THF), 2-MeTHF, 1,4-dioxane, acetone and MeCN. Moreover, immobilized RML was very thermostable up to 50 °C. It is noteworthy that immobilized RML and MJL showed high activity over 30 cycles and maintained the same initial enantioselectivity. RML immobilized in micro-emulsion-based organogels was employed for a 5-*g*-scale kinetic resolution of Ketoprofen vinyl ester, furnishing the desired product (91% *ee*) in 47% yield after 72 h (Scheme 10b and Table 1). The authors highlight the advantages of biotransformations catalyzed by immobilized enzymes in the form of MBGs; these advantages include an improved enzymatic efficiency (conversion and enantioselectivity), excellent reusability, low enzyme loading, and enhanced resistance to organic solvents. Long-term resistance to high concentrations of organic solvents is a crucial feature in lipase-catalyzed reactions, especially when working with poorly water-soluble substrates. Studies on the effect of interfacial composition on lipase activity in two-liquid phase systems revealed that substrate inaccessibility is a major reason for the low activity of lipase in the absence of organic solvents [23]. 

**Table 1:** Enzymes and conditions used on the kinetic enzymatic hydrolysis of *rac*-Ketoprofen vinyl ester by methods a and b.

Method	Enzyme	Conditions	Reference
a	lipase from *A. terreus* immobilized on Alg-*g*-PEG/α-CD, hollow spheres	acetone/water (80/20, *v*/*v*), pH 7.4, 30 °C, 4 days	[21]
b	MJL MBGs or RML MBGs	DIPE, 30 °C, 72 h	[22]

### 2.10. Dihydropyridine Derivatives

**Scheme 11:**
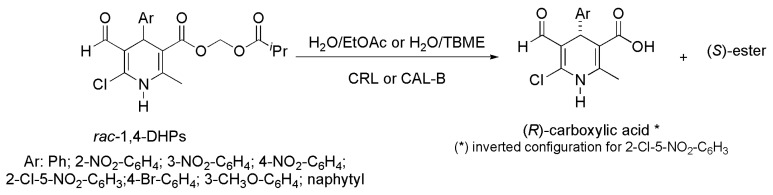
Kinetic enzymatic hydrolysis of racemic 1,4-DHPs to obtain the chiral pharmacophores used in clinics for the treatment of cardiovascular diseases [24].

The 1,4-Dihydropyridines (1,4-DHPs) are recognized as pharmacophores widely used in clinics for the treatment of cardiovascular diseases. A series of racemic 1,4-DHPs were prepared through several chemical steps including a multicomponent Hantzsch process and a Vilsmeir-Haack reaction. These 1,4-DHPs were subjected to enzymatic kinetic resolution via hydrolysis in the presence of lipases in water saturated with organic solvent [24]. Reactions were carried out until conversions next to 50% and several lipases (such as from *Candida antarctica* A (CAL-A, NZL-101), porcine pancreatic lipase (PPL), from *C. antarctica* B (CAL-B, Novozym 435) and from *C. rugosa* (CRL, type VII)) were assayed. The best results were obtained in the presence of CRL or CAL-B depending on the aryl group and the organic solvent. For reaction systems containing CRL and EtOAc as a solvent, the highest enantioselectivity values (*E*) were obtained with 1,4-DHPs containing aryl groups as 2-NO_2_–C_6_H_4_, naphtyl and 2-Cl–5-NO_2_–C_6_H_3_ with *E*-values of 103, 170 and >200, respectively. The *ee* values of the remaining ester were in the range of 84% to >99% while the *ee* of the carboxylic acid product ranged from 94% to 98%. It is noteworthy that some of the carboxylic acid products had the enantiomeric excess increased by crystallization. Applying this methodology, *ee* values of the carboxylic acid products containing aryl groups (*i.e.*, 3-NO_2_–C_6_H_4_; Ph; and 4-NO_2_–C_6_H_4_) were enhanced from 88% to 97%; 60% to 92%; and 69% to 95%, respectively. In the presence of CAL-B and EtOAc as a solvent, the best result was obtained with a 1,4-DHP-containing aryl group such as 3-CH_3_O–C_6_H_4_ with an *E*-value of 63, c 34%, 49% *ee* to the remaining ester and 95% *ee* to the carboxylic acid product. In order to obtain the remaining substrate with high *ee*, hydrolysis of 1,4-DHPs containing aryl groups such as 4-Br–C_6_H_4_ and 3-CH_3_O–C_6_H_4_ was carried out with CAL-B and TBME as a solvent affording 57% and 54% molar conversion, respectively. In this case, remaining substrates (aryl = 4-Br–C_6_H_4_) and (aryl = 3-CH_3_O–C_6_H_4_) were obtained with high *ee*, 97% and 94%, respectively (Scheme 11).

## 3. Esterification Approach

As known to most of the researchers working in this area, lipases can efficiently catalyze the stereoselective acylation of alcohols when the reaction is performed under low water activity conditions. This opportunity can be exploited for achieving the highly regio- or stereoselective preparation of esters with pharmaceutical relevance under mild and controlled conditions. Examples span from structurally simple drugs, such as nonsteroidal anti-inflammatory drugs (NSAIDs), to multifunctional complex molecules, such as immunosuppressive agents (*i.e.*, Rapamycin or Pimecrolimus), where regioselectivity is a major issue.

### 3.1. Rapamycin

**Scheme 12:**
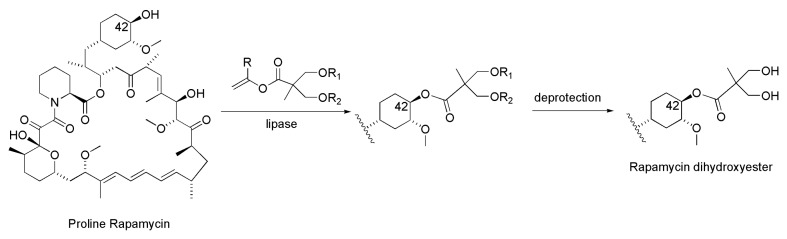
Chemoenzymatic synthesis of proline analogue of Rapamycin dihydroxyester [25].

A brilliant example of regioselectivity observed in lipase-catalyzed acylation is the synthesis of the proline analogue of Rapamycin dihydroxyesters, where the chemoenzymatic approach allowed the preparation of the desired molecule in only two steps [25]. Identification of a suitable activated ester of the 2,2-bis(hydroxymethyl) propionic acid side chain was crucial for performing the lipase-catalyzed acylation; vinyl esters provided the highest activity and the best yield, allowing for preparative synthesis. Immobilized lipase PS-C “Amano” II was the best biocatalyst, displaying optimal activity in anhydrous TBME (Scheme 12).

### 3.2. Pimecrolimus

**Scheme 13:**
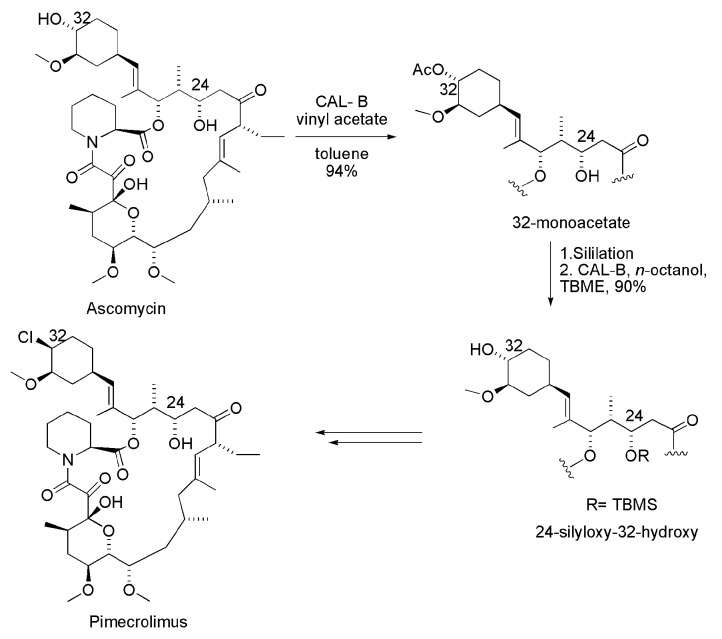
Synthesis of Pimecrolimus via regioselective enzymatic acylation of Ascomycin [26,27].

Pimecrolimus (32-*epi*-chloro-derivative of Ascomycyn) is a macrolide which has anti-inflammatory, antiproliferative and immunosuppressive properties, used in the topical treatment of inflammatory skin diseases. This substance is present as an active ingredient in the drug marketed as Elidel^®^. The first chemoenzymatic synthesis of Pimecrolimus was performed from Ascomycin, a substance isolated from the fermentation broth of *Streptomyces hygroscopicus*. Ascomycin has two hydroxyl groups attached to secondary carbons, one in the cyclohexane moiety (C-32) and the other in macrolactam cycle (C-24). In this synthesis, it was necessary to acetylate the hydroxyl specifically located in cyclohexane moiety. The strategy was based on the regioselectivity of lipase from *Candida antarctica* B (CAL-B, Novozym 435) with regioselective acylation of the hydroxyl group in question, in the presence of vinyl acetate and toluene, leading to the corresponding acetate with 94% yield (Scheme 13). Then, the 32-monoacetate derivative was subjected to silylation with trimethylsilyl trifluoromethanesulfonate (TBMSOTf), leading to a 24-silyloxy-32-monoacetate derivative. The latter was subjected to a regioselective alcoholysis (*n*-octanol) in the presence of a CAL-B-producing 24-silyloxy-32-hydroxy derivative. After the steps of introducing the chlorine atom at position 32 with polymer-bound triphenyl phosphine and deprotection of the silyl group at position 24, Pimecrolimus was obtained with an overall yield of 29% [26,27].

### 3.3. Loxoprofen

**Scheme 14:**
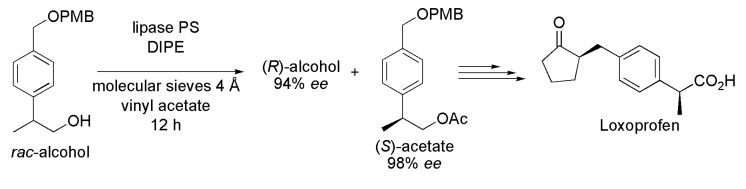
Enzymatic kinetic resolution of the racemic alcohol 2-(*p*-{[(*p*-methoxy phenyl) methoxy]methyl}phenyl)propanol to produce the (*S*)-acetate used in the synthesis of the drug Loxoprofen [28].

A considerable number of biocatalytic routes have been developed so far for the production of (*S*)-profens; nevertheless, more efficient and sustainable processes are needed due to the commercial importance of these drugs. Loxoprofen, an (*S*)-2-(4-(*R*)-2-oxo–cyclopentylmethyl)phenyl propionic acid, is a NSAI drug belonging to the group of propionic acid derivatives with anti-inflammatory, analgesic and antipyretic activities. In fact, Loxoprofen is a pro-drug because its active form is the corresponding *trans*-alcohol metabolite. The preparation of Loxoprofen involved the enzymatic kinetic resolution of the racemic alcohol 2-(*p*-{[(*p*-methoxy phenyl)methoxy]methyl}phenyl)propanol via transesterification reaction in the presence of lipase from *Burkholderia cepacia* (lipase-PS), molecular sieves 4 Å, in DIPE as a solvent and vinyl acetate as an acyl donor (Scheme 14). After 12 h, it was possible to obtain the corresponding (*S*)-acetate in 98% enantiomeric excess (*ee*) and the (*R*)-alcohol in 94% *ee*. The synthesis of Loxoprofen followed with (*S*)-acetate having the desired configuration of the drug [28].

### 3.4. Flurbiprofen

**Scheme 15:**
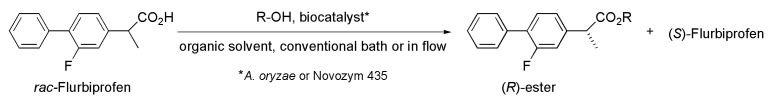
Enzymatic kinetic resolution of racemic Flurbiprofen, a non-steroidal anti-inflammatory drug, using classical batch method [29] and inflow reactors [30,31].

(*S*)-Flurbiprofen is a NSAI drug, whereas its enantiomer was found to inhibit tumor growth in animal models. The kinetic resolution of racemic Flurbiprofen was performed using dry mycelia of *Aspergillus oryzae*, MIM as biocatalysts [29]. After optimizing the reaction conditions (organic solvent, type of alcohol and temperature), (*R*)-Flurbiprofen ester was obtained in varied conversion and enantioselectivity (62%–92% *ee*), as seen in Scheme 15. Later on, the kinetic resolution of the same racemic drug was performed in flow-reactor, which is considered an interesting approach for the development of the lipase-catalyzed preparation of enantiopure molecules [30,31]. It is also worth mentioning that these automated systems help continuous bioprocesses, provide easy reaction optimization (including parameters such as residence time), and allow for multistep reactions and inline recovery and purification of the products [32,33]. Thus, as a proof of concept, direct esterification in organic solvent using commercial enzyme (Novozym 435) [30] and the whole microorganism (*A. oryzae*, MIM) [31] were compared with the classical batch method. The protocol inflow reactor showed a significant reduction of the reaction time (from 6 h to 15–60 min) and yielded both the (*S*)-Flurbiprofen and (*R*)-Flurbiprofen butyl ester with ≥90% *ee* (chemical purity >98%) by modulating the reaction conditions (temperature and residence time), as seen in Scheme 15.

### 3.5. Ibuprofen

**Scheme 16:**
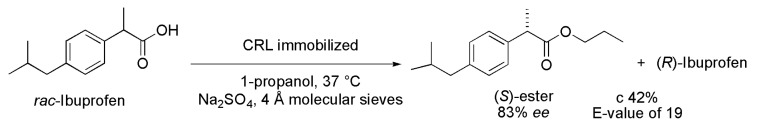
Kinetic enzymatic esterification of *rac*-Ibuprofen, a nonsteroidal drug with anti-inflammatory activity [34].

Ibuprofen is another NSAI agent with anti-inflammatory activity and wide commercial interest. A racemic mixture of Ibuprofen was resolved in the presence of commercially available lipases OF and CRL type VII from *Candida rugosa* immobilized onto magnetic beads. Immobilization was performed via a glutaraldehyde or *N*-3-(3-dimethylaminopropyl)-*N*ߢ-ethylcarbodiimide (EDC)/*N*-hydroxysulfosuccinimide sodium salt (sulfo-NHS) cross-linking reaction. The immobilized enzymes were evaluated for the preparation of Ibuprofen propyl ester. All reactions provided the (*S*)-ester as the product, and the best result (*E*-value of 19, 83% *ee* and c 42%) was achieved in the presence of the immobilized lipase from *C. rugosa* OF, as well as in the presence of additives such as Na_2_SO_4_ and molecular sieves 4 Å (Scheme 16). The study of reuse demonstrated continued stability and catalytic activity after five reaction cycles [34].

### 3.6. Ketorolac

**Scheme 17:**
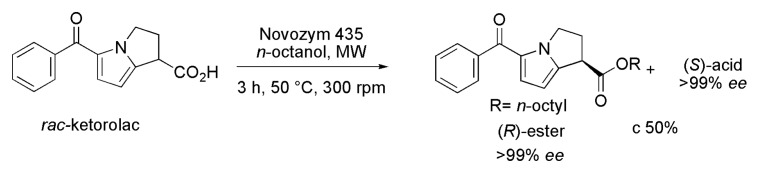
Enzymatic kinetic resolution of *rac*-Ketorolac, a potent nonsteroidal anti-inflammatory drug [35].

Ketorolac (*rac*-5-benzoyl-1,2-dihydro-3*H*-pyrrolo[1,2-*a*]pyrrole-1-carboxylic acid) is a potent NSAI drug belonging to the class of the heterocyclic acetic acid derivatives, and it is widely used as an analgesic. The microwave (MW)-assisted kinetic resolution of this compound was investigated using different immobilized lipases, such as Novozym 435, Lipozyme TL IM, Lipozyme RM IM, lipase Amano AS, and lipase AYS Amano [35]. Among them, Novozym 435 catalyzed the enantioselective acylation of *rac*-Ketorolac in 3 h, at 50 °C and 300 rpm, with c 50% and high *ee* values (>99%) for both the (*R*)-ester and remaining (*S*)-acid (Scheme 17). In addition, the reaction was found to follow the Ping-Pong bi-bi mechanism, and to be inhibited by *n*-octanol.

### 3.7. Chloramphenicol and Thiamphenicol

**Scheme 18:**
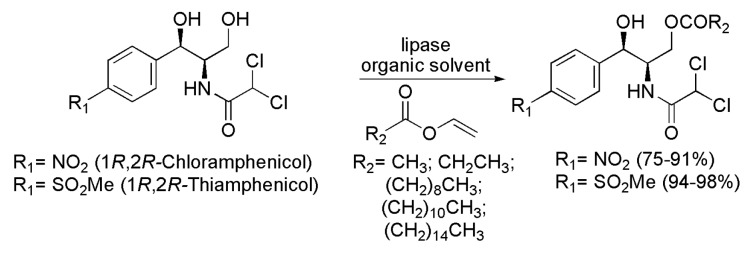
Regioselective enzymatic acylation of (−)-Chloramphenicol [36] and (−)-Thiamphenicol [37].

Another pharmacological field where the use of lipases has been particularly active in the recent past is the elaboration of antibiotics. The compound (1*R*,2*R*)-(−)-Chloramphenicol is an effective antibiotic against a wide range of Gram-positive and Gram-negative bacteria. This drug can be administered in capsule or liquid forms, and it has a bitter taste. One way to overcome this problem is to use esters of chloramphenicol, which are more palatable. Several chloramphenicol esters were prepared regioselectively in the presence of lipases. Then (−)-Chloramphenicol was subjected to esterification reaction in the presence of a series of vinyl esters containing alkyl chains of varying sizes (one to 15 carbon atoms), as well as in the presence of a number of lipases. The more efficient lipases were from *Candida antarctica* B (CAL-B, Novozym 435) and two different preparations from *Pseudomonas cepacia*, one from Amano (PSL-C Amano, also known as *Burkholderia cepacia*) and another from Sigma-Aldrich^®^ (PSLC-I). With CAL-B, the best reaction conditions were in the presence of MeCN as a solvent at 20 °C; with PSL-C I and PSL-C Amano, the ideal solvent was 1,4-dioxane at 30 °C. Generally, monoesters of chloramphenicol acylated on the primary hydroxyl were obtained with c >99% (isolated yields between 75% and 91%) for reaction times between 3 and 10 h (Scheme 18 and Table 2). It is noteworthy that the study of the reuse of CAL-B in esterification with vinyl palmitate was performed. As a result, it was observed that after 3 h of reaction, there was complete conversion during 10 cycles of reuse of the enzyme [36]. The same strategy was used on the synthesis of Thiamphenicol esters from (1*R*,2*R*)-(−)-Thiamphenicol, an antibiotic analogue of chloramphenicol with veterinary applications. The esters were regioselectively prepared via acylation reaction in the presence of lipases and vinyl esters with variable lengths. The most effective lipase was CAL-B in MeCN, at 20 °C. Thiamphenicol esters (acylated at the primary hydroxyl group) were regioselectively produced with c >99% and isolated yields between 94% and 98%, as seen in Scheme 18 and Table 2. A study of the reuse of lipase from *C. antarctica* type B (CAL-B, Novozym 435) was conducted for the acylation of (−)-Thiamphenicol with vinyl decanoate. The corresponding 3’-monoester was obtained in 96% yield and the enzyme was reused five times without any loss of the activity [37].

**Table 2:** Optimized conditions for the regioselective enzymatic acylation of (−)-Chloramphenicol and (−)-Thiamphenicol.

Compound	Enzyme	Conditions	3‘-Monoester Yield (%)	Ref.
(−)-Chloramphenicol	CAL-B PSL-C I or PSL-C Amano	MeCN, 20 °C 1,4-dioxane, 30 °C	75–91	[36]
(−)-Thiamphenicol	CAL-B	MeCN, 20 °C	94–98	[37]

### 3.8. Key Intermediates of Modified Cephalosporins

**Scheme 19:**
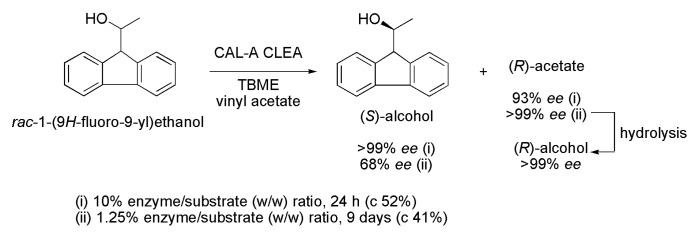
Kinetic enzymatic acylation of *rac*-1-(9*H*-fluoren-9-yl)ethanol, affording intermediates in the synthesis of modified Cephalosporins and the anti-inflammatory agent *N*-[(9*H*-fluoren-9-ylethoxy)carbonyl]-l-leucine *tert*-butyl ester [38].

Some modified Cephalosporins and the anti-inflammatory agent *N*-[(9*H*-fluoren-9-ylethoxy)carbonyl]-l-leucine *tert*-butyl ester have a chiral fragment resulting from the incorporation of 1-(9*H*-fluoren-9-yl)ethanol in their structures. The first chemoenzymatic synthesis of this chiral fragment was performed in the presence of lipases. Initially, racemic 1-(9*H*-fluoren-9-yl)ethanol was chemically obtained, and then it was submitted to a kinetic resolution via acetylation reaction. Fifteen commercial lipases were evaluated by studying parameters considered important in the kinetic resolution process, such as the substrate/lipase ratio and solvent. The best results were obtained in the kinetic resolution with an enzymatic aggregate of lipase from *Candida antarctica* A (CAL-A CLEA, Amano lipase A). It is worth noting that depending on the reaction conditions, both (*S*)- and (*R*)-1-(9*H*-fluoren-9-yl) ethanol (obtained from the hydrolysis of the corresponding acetate) could be prepared with >99% *ee*. In the first condition (i), after 24 h of reaction, with an enzyme/substrate (*w*/*w*) ratio of 10% and TBME as a solvent, it was possible to obtain the (*S*)-alcohol and (*R*)-acetate with enantiomeric excess (*ee*) >99% and 93%, respectively (*E*-value of 145, c 52%), as seen in Scheme 19. In the second condition (ii), a dramatic decrease in the reaction rate was observed with a decreasing enzyme/substrate ratio to 1.25%. After nine days, the (*S*)-alcohol (68% *ee*) and (*R*)-acetate (>99% *ee*) were obtained with c 41% and *E* > 200 [38].

### 3.9. Key Intermediate of Duloxetin

**Scheme 20:**
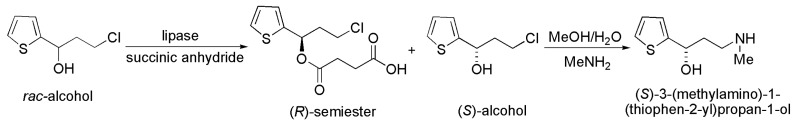
Kinetic enzymatic acylation of the *rac*-3-chloro-1-(thiophen-2-yl)propan-1-ol used in the chemoenzymatic synthesis of (*S*)-3-(methylamino)-1-(thiophen-2-yl)propan-1-ol, a key intermediate in the synthesis of the serotonin-norepinephrine reuptake inhibitor Duloxetine [39].

BASF AG patented the chemoenzymatic synthesis of (*S*)-3-(methylamino)-1-(thiophen-2-yl)propan-1-ol, a key intermediate in the synthesis of the serotonin-norepinephrine reuptake inhibitor Duloxetine ((+)-(*S*)-*N*-methyl-3-(1-naphthyloxy)-3-(2-thienyl)propylamine oxalate) [39]. Chemical reduction of 3-chloro-1-(thiophen-2-yl)propan-1-one gives access to the corresponding *rac*-alcohol, which was enantioselectively acylated with succinic anhydride using lipase from *Burkholderia plantarii* or from *Pseudomonas* sp., furnishing the corresponding (*R*)-semiester, leaving the unreacted (*S*)-alcohol. The reaction between the (*S*)-alcohol and methylamine afforded the enantiomerically pure desired product (Scheme 20).

### 3.10. Nebracetam

**Scheme 21:**
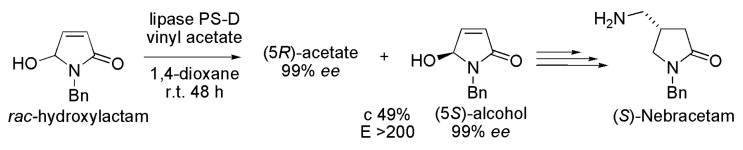
Kinetic resolution of the racemic hydroxylactam to produce the (5*S*)-1-benzyl-5-hydroxy-1,5-dihydropyrrol-2-one used in the synthesis of the drug (*S*)-Nebracetam [40].

Nebracetam, a nootropic drug from the racetam family, is used as an anti-depressant (M1 acetylcholine receptor agonist). The first asymmetric synthesis of (*S*)-Nebracetam was efficiently conducted from the intermediate (5*S*)-1-benzyl-5-hydroxy-1,5-dihydropyrrol-2-one which was obtained by kinetic resolution of the racemic hydroxylactam (1-benzyl-5-hydroxy-1,5-dihydropyrrol-2-one). The acetylation was performed in the presence of lipase from *Burkholderia cepacia* (lipase PS-D), vinyl acetate as an acyl donor, 1,4-dioxane as the organic solvent at room temperature and 48 h of reaction (Scheme 21). Accordingly, the corresponding (*R*)-acetate and (*S*)-alcohol were obtained with excellent enantioselectivity and conversion (>99% *ee*, c 49% and *E* > 200). Then, after several chemical steps, the (5*S*)-alcohol was converted into (*S*)-Nebracetam [40].

### 3.11. Naphthofurandione Derivative

**Scheme 22:**
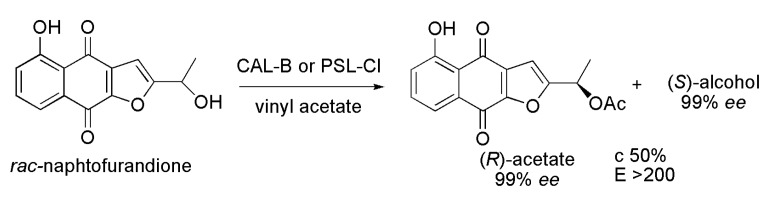
Synthesis of the naphthofurandione (*S*)-5-hydroxy-2-(1-hydroxyethyl)naphtho[2,3-*b*]furan-4,9-dione via kinetic enzymatic acylation of the corresponding racemic alcohol [41].

The naphthofurandione (*S*)-5-hydroxy-2-(1-hydroxyethyl)naphtho[2,3-*b*]furan-4,9-dione, with antitumor activity, is a secondary metabolite isolated from the bark of *Tabebuia impetiginosa* (Bignoniaceae). Naphthofurandione was prepared in racemic form through several chemical steps from commercially available 1,5-dihydroxynaphthalene. Then, the *rac*-naphthofurandione was subjected to enzymatic kinetic resolution via acetylation reaction (Scheme 22). Among the evaluated lipases, *Pseudomonas cepacia*, (PSL-CI, 3 h of reaction) and lipase from *Candida antarctica* B, (Novozym 435, 2 h of reaction) were the most efficient. The best reaction conditions were vinyl acetate as the acyl donor, THF as the solvent, 250 rpm, at 30 °C, leading to (*S*)-alcohol and the corresponding (*R*)-acetate with 99% *ee* for both and *E* > 200 [41].

### 3.12. GABOB and Carnitine

**Scheme 23:**
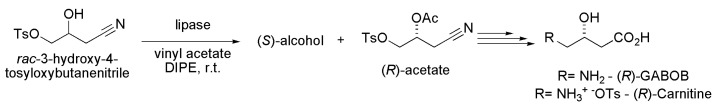
Kinetic resolution of the racemic 3-hydroxy-4-tosyloxybutanenitrile to produce (*R*)-GABOB and (*R*)-carnitine [42].

The compounds (*R*)-γ-Amino-β-hydroxybutyric acid (GABOB) and (*R*)-carnitine are two important bioactive molecules that play an important role in mammalian systems. The key step of the chemoenzymatic syntheses of these two compounds involved the lipase-mediated resolution of the racemic 3-hydroxy-4-tosyloxybutanenitrile in organic solvents (Scheme 23). Lipases (free or immobilized forms) from *Pseudomonas cepacia* (PS), *P. fluorescens* (AK), *Candida rugosa* (CRL AYS), *C. antarctica* (CAL-B) and *Mucor miehei* (Lipozyme) were used as biocatalysts, and yielded the (*S*)-alcohol and (*R*)-acetate in high enantioselectivity (>99% *ee*). After some chemical transformations, the (*R*)-acetate isomer was converted into (*R*)-GABOB and (*R*)-carnitine [42].

### 3.13. Quinolone Derivatives

**Scheme 24:**
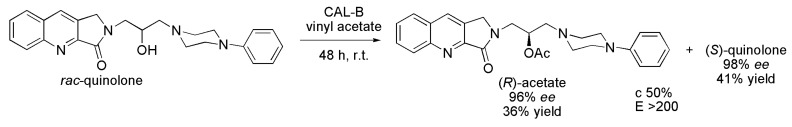
Kinetic enzymatic acylation of the racemic quinolone 2-[2-hydroxy-3-(4-phenylpiperazin-1-yl)propyl]-1*H*-pyrrolo[3,4-*b*]quinolin-3(2*H*)-one [43].

The quinolone 2-[2-hydroxy-3-(4-phenylpiperazin-1-yl)propyl]-1*H*-pyrrolo[3,4-*b*]quinolin-3(2*H*)-one was chemically synthesized and subsequently subjected to enzymatic kinetic resolution via acylation reaction (Scheme 24). The best results were obtained using lipase from *Candida antarctica* B (CAL-B), and vinyl acetate as a both solvent and acyl donor at room temperature. After 48 h of reaction, it was possible to obtain the corresponding (*R*)-acetate in 36% yield and 96% *ee*; (*S*)-quinolone was obtained in 41% yield and 98% *ee*, c 50% and *E* > 200. The (*S*)-quinolone as well as the corresponding (*R*)-acetate showed significant cytotoxic activity against human neuroblastoma tumor cells (SK-N-SH) and lung cells (A549) compared to the control doxorubicin [43].

### 3.14. Key Intermediate of Mevinic Acid Analogues

**Scheme 25:**
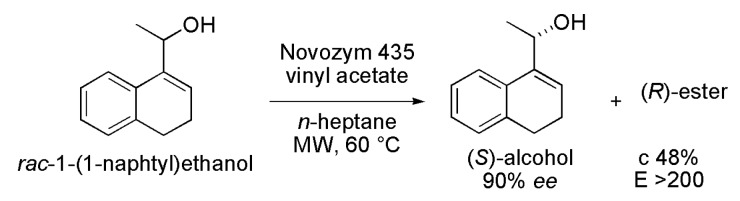
Kinetic enzymatic acylation of *rac*-1-(1-naphthyl)ethanol to produce the (*S*)-1-(1-naphthyl)ethanol, an intermediate in the synthesis of mevinic acid analogues [44].

Analogues of mevinic acid can be obtained from (*S*)-1-(1-naphthyl)ethanol. A racemic mixture of this compound was resolved in the presence of lipases using microwave. Several parameters were studied and the best reaction conditions were Novozym 435 as a biocatalyst, vinyl acetate as an acyl donor, *n*-heptane as a solvent, a stirring speed of 400 rpm, temperature at 60 °C and 30 mg of enzyme loading. In such conditions, after 3 h of reaction, the (*S*)-1-(1-naphthyl)ethanol was obtained with c 48%, 90.0% *ee* and *E* > 200 (Scheme 25). The study of reuse of the enzyme was performed with a slight reduction of conversion from 48% to 45% after being used three times. It is noteworthy that the results from the conventional heating (c 39%, 64% *ee* and *E*-value of 164 after 5 h of reaction) were inferior to those obtained under microwave conditions [44].

### 3.15. Key Intermediate of Tetrahydrofuran Derivatives

**Scheme 26:**
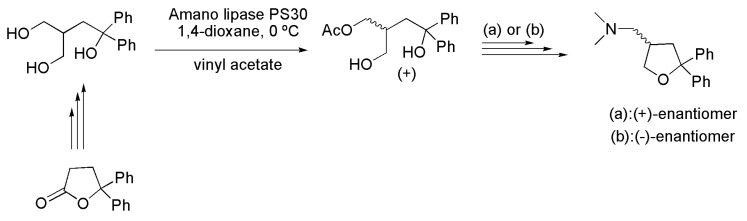
Desymmetrization of the intermediate 3-(hydroxymethyl)-1,1-diphenylbutane-1,4-diol to produce (+)-4-hydroxy-2-(hydroxymethyl)-4,4-diphenylbutyl acetate, a key intermediate in the synthesis of (+)- and (−)-1-(5,5-diphenyltetrahydrofuran-3-yl)-*N*,*N*-dimethylmethanamine [45].

Compounds (+)- and (−)-1-(5,5-diphenyltetrahydrofuran-3-yl)-*N*,*N*-dimethylmethanamine showed neuroprotective, antidepressant and antiepileptic activities. Such compounds were prepared by chemoenzymatic synthesis starting from 5,5-diphenyltetrahydrofuran-2(3*H*)-one. The key step for introducing chirality in the target molecule was the desymmetrization of the intermediate 3-(hydroxymethyl)-1,1-diphenylbutane-1,4-diol, using vinyl acetate as an acylating agent in the presence of lipase from *Burkholderia cepacia* (Amano lipase PS30), to obtain the (+)-4-hydroxy-2-(hydroxymethyl)-4,4-diphenylbutyl acetate (Scheme 26). The latter compound was subjected to two different synthetic routes. The first (a) involved a sequence of reactions including tosylation, and hydrolysis and reaction with 2,6-lutidine in the presence of trifluoromethane sulfonic anhydride to afford the (+) 1-(5,5-diphenyltetrahydrofuran-3-yl)-*N*,*N*-dimethylmethanamine. In the second synthetic route (b), the intermediate (+)-4-hydroxy-2-(hydroxymethyl)-4,4-diphenylbutyl acetate was subjected to a step of protecting one of the hydroxyl groups with *tert*-butyldimethylsilyl chlroride (TBSCl), followed by hydrolysis in the presence of LiOH/H_2_O, tosylation reaction and deprotection with tetrabutylammonium fluoride (TBAF). Finally, a reaction with 2,6-lutidine in the presence of trifluoroacetic sulfonic anydride led to the (−)-1-(5,5-diphenyltetrahydrofuran-3-yl)-*N*,*N*-dimethylmethanamine. It should be mentioned that the absolute configurations of the products were not reported by the authors [45].

### 3.16. Key Intermediates of β-Amino Alcohols

**Scheme 27:**
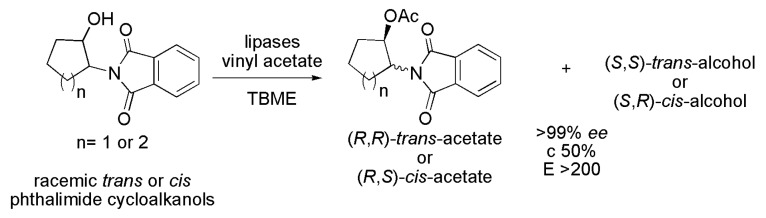
Kinetic enzymatic acylation of racemic *trans*- and *cis*-diastereoisomers of 2-phthalimidocyclopentanol and 2-phthalimidocyclohexanol to produce intermediates in the synthesis of β-amino alcohols [46].

β-Amino alcohols are part of the structure of numerous active pharmaceuticals such as Vernakalant and Indinavir. The *cis*- and *trans*-2-phthalimidocyclopentanol and 2-phthalimidocyclohexanol were subjected to kinetic resolution in the presence of lipases. The best reaction conditions were obtained in the presence of vinyl acetate and TBME. For both *trans*-2-phthalimidocyclopentanol and *trans*-2-phthalimidocyclohexanol the most efficient lipase was from *Pseudomonas cepacia* (PS IM), as seen in Table 3, leading to (1*R*,2*R*)-acetates and (1*S*,2*S*)-alcohols with >99% *ee*, c 50% and *E* > 200. In this case, the biocatalyst maintained its activity and enantioselectivity after five reaction cycles. For the *cis*-2-phthalimidocyclopentanol, four lipases were efficient: lipase A from *Candida antarctica* (CAL-A), lipase from *Rhizomucor miehei* (RM IM), lipase from *P. fluorescens* (AK) and *P. cepacia* lipase (PS IM), as seen in Table 3. In all cases (1*S*,2*R*)-*cis*-alcohol and (1*R*,2*S*)-*cis*-acetate with >99% *ee*, c 50%, and *E* > 200 were obtained. For the *cis*-2-phthalimidocyclohexanol the only effective lipase was *P. cepacia* (PS IM), Table 3, with a reaction time of 72 h, at 45 °C. In such conditions, (1*R*,2*S*)-*cis*-acetate and (1*S*,2*R*)-*cis*-alcohol were obtained with >99% *ee*, c 50% and *E* > 200 (Scheme 27) [46].

**Table 3:** Efficient lipases for the kinetic enzymatic acylation of racemic *trans*- and *cis*-diastereoisomers of 2-phthalimidocyclopentanol and 2-phthalimidocyclohexanol [46].

Compound	Lipase
*trans*-2-phthalimidocyclopentanol	*P. cepacia* (PS IM)
*cis*-2-phthalimidocyclopentanol	*C. antarctica* (CAL-A), *R. miehei* (RM IM), *P. fluorescens* (AK) and *P. cepacia* (PS IM)
*trans*-2-phthalimidocyclohexanol	*P. cepacia* (PS IM)
*cis*-2-phthalimidocyclohexanol	*P. cepacia* (PS IM)

### 3.17. Key Intermediates of Iminocyclitols

**Scheme 28:**
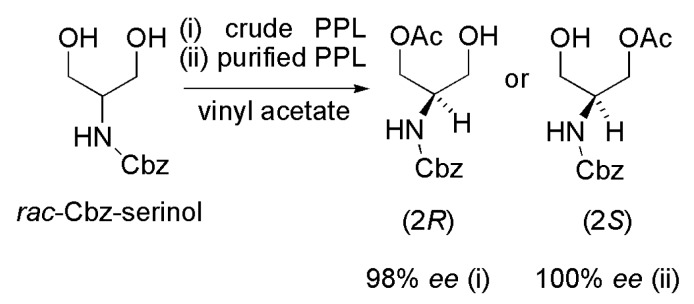
Kinetic enzymatic acylation of racemic carboxybenzyl-2-amino-1,3-propanediol (Cbz-serinol) to produce intermediates in the synthesis of iminocyclitols [47].

Enantiomerically pure 1,3-propanediols are important intermediates in the synthesis of iminocyclitols (glycosidases inhibitors used as anti-diabetic drugs). The desymmetrization of carboxybenzyl-2-amino-1,3-propanediol (Cbz-serinol) via acetylation reaction in THF mediated by crude pig pancreatic lipase (PPL, L3126, type II, Steapsin) (i) or purified PPL (L0382, type VI-S) (ii) was reported [47]. When the reaction was carried out in vinyl acetate as both a solvent and acyl donor, in the presence of crude PPL (i), only the (2*R*)-monoacetate was formed in 98% *ee*. On the other hand, only the (2*S*)-monoacetate (100% *ee*) was formed when purified PPL (ii) catalyzed the desymmetrization (Scheme 28).

### 3.18. N-Acetyl Phenylalanine and Analogues

**Scheme 29:**
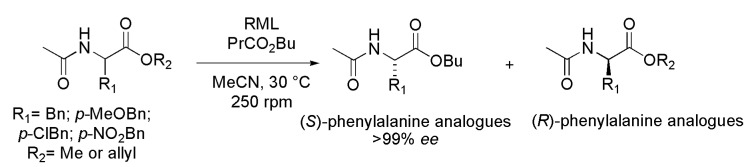
Kinetic enzymatic resolution of racemic *N*-acetyl phenylalanine and analogues via interesterification reaction [48].

Phenylalanine and analogues are important building blocks in the preparation of complex structures of interest to the medicinal chemistry area. *N*-Acetyl phenylalanine and a series of analogues were obtained in enantiomerically pure form by interesterification reaction in the presence of lipase from *Rhizomucor miehei* (RML). Ethyl acetamidocianoacetate was alkylated in the presence of various alkyl halides in phase transfer catalysis (PTC), followed by acidic hydrolysis, esterification and *N*-acetylation leading to *N*-acetyl-phenylalanine methyl and allyl esters derivatives. These derivatives were submitted to an enzymatic kinetic resolution via interesterification reaction. After screening with various commercial lipases, it was found that only RML was able to promote the kinetic resolution (Scheme 29). Several parameters were studied and the best reaction conditions were obtained in the presence of butyl butyrate as an interesterification agent, with an enzyme:substrate ratio of 2:1 (*w*/*w*), at 30 °C, and MeCN as a solvent. Most derivatives (*S*-configuration) were obtained with >99% *ee* and *E* > 200 [48].

### 3.19. (S)-1-(2-Furyl)ethanol

**Scheme 30:**
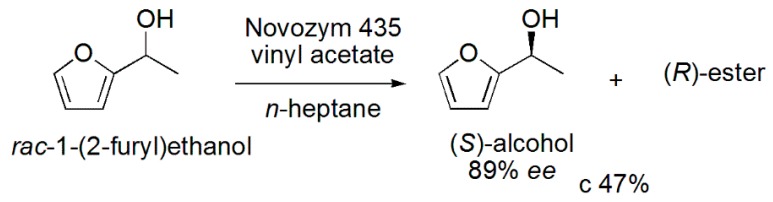
Kinetic enzymatic acylation of the *rac*-1-(2-furyl)ethanol to produce the (*S*)-alcohol, an important building block for the synthesis of various natural products [49].

The (*S*)-1-(2-furyl)ethanol is used as an important building block for the synthesis of various natural products such as flavonoids, polyketide antibiotics and carbohydrate derivatives. This intermediate was obtained by lipase-catalyzed kinetic resolution of racemic 1-(2-furyl)ethanol. After commercial lipases screening in *n*-heptane as a solvent and 2 h of reaction time, the lipase from *Candida antarctica* B (Novozym 435) was the most efficient enzyme, leading to higher conversion (c 47%) than those observed with the other evaluated enzymes, RM IM Lipozyme (c 1.8%) and Lipozyme TL IM (c 1.5%). Various parameters were studied before establishing the best reaction conditions (vinyl acetate as the acyl donor, *n*-heptane as the solvent, a stirring speed of 300 rpm, 5 mg of enzyme loading at 60 °C). In these conditions, the (*S*)-1-(2-furyl)ethanol was obtained with c 47% and 89% *ee* (Scheme 30). The study of the reuse of the biocatalyst was also performed, and it was shown that it can be recycled three times with a small decrease in conversion from 47.0% to 44.5% [49].

### 3.20. Ascorbyl Ester Derivatives

**Scheme 31:**
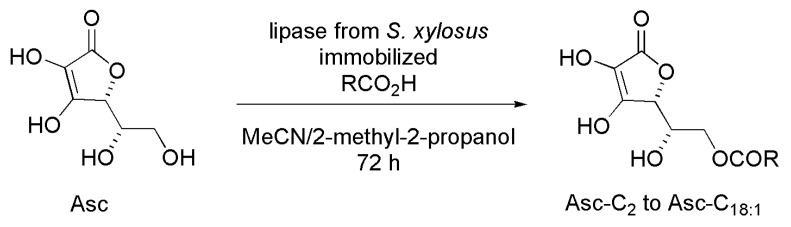
Kinetic enzymatic acylation of the ascorbic acid (Asc) to produce ascorbyl esters derivatives [51].

Ascorbic acid (Asc) is a very important natural antioxidant, but with limited industrial application because of its hydrophilic nature. Alternatively, ascorbyl esters are considered antioxidants to be used in hydrophobic formulations [50]. A series of lipophilic esters derived from ascorbic acid was prepared by using lipase from *Staphylococcus xylosus* immobilized on silica aerogel. The regioselective enzymatic esterification of Asc with seven carboxylic acids (acetic acid and six fatty acids) was performed in MeCN and 2-methyl-2-propanol (co-solvent) for 72 h. Yields varied with the length of the fatty acid chain, and the best result was obtained on the preparation of the short chain derivative ascorbyl acetate (82.6 % yield), as seen in Scheme 31. All Asc derivatives were submitted to some bioassays (antibacterial, antioxidant, and antileishmanial), and the results suggested them as candidates to be used in the cosmetic and pharmaceutical industries [51].

### 3.21. Key Intermediates of Stagonolide E, Pyrenophorol and Decarestrictine L

**Scheme 32:**
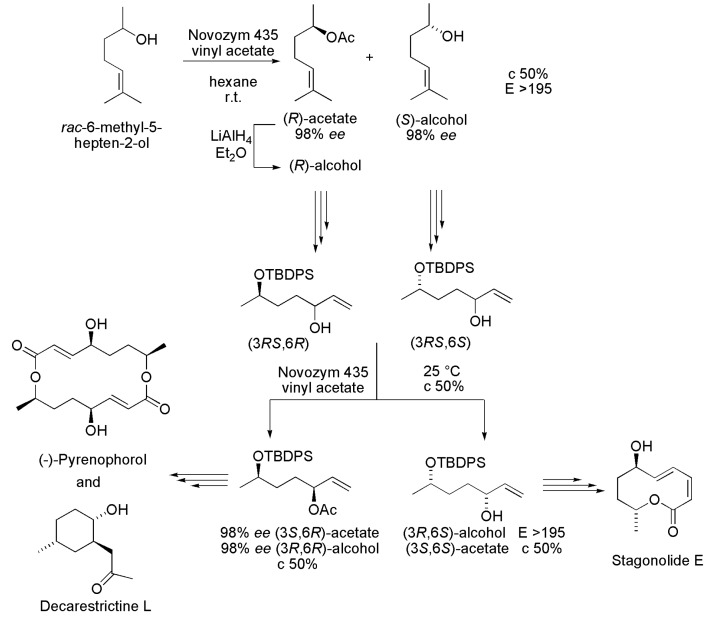
Kinetic enzymatic acylation of *rac*-hept-6-ene-2,5-diol derivatives to produce key intermediates in the synthesis of the Stagonolide E, Pyrenophorol and Decarestrictine L [52].

Enantiomerically pure hept-6-ene-2,5-diol derivatives were prepared by lipase-catalyzed acetylation of the corresponding racemates, and used as key intermediates in the synthesis of the bioactive compounds Stagonolide E, Pyrenophorol and Decarestrictine L (Scheme 32), all of them isolated from filamentous fungi. Racemic 6-methyl-5-hepten-2-ol was resolved by acetylation with Novozym 435, vinyl acetate in hexane at room temperature, and yielded both the (*R*)-acetate and (*S*)-alcohol with 98% *ee* (*E* > 195) after 50% conversion. The (*R*)-alcohol was also produced by treatment of (*R*)-acetate with LiAlH_4_. Then, both the (*R*)- and (*S*)-alcohols were submitted, individually, to some chemical steps that included protection of the hydroxyl group with *tert*-butyldiphenylsilyl (TBDPS), ozonolysis and reaction of the obtained aldehydes with vinylmagnesium bromide, providing the allylic alcohols (3*RS*,6*R*) and (3*RS*,6*S*). A similar protocol was used in the enzyme-catalyzed acylation of these allylic alcohols (Scheme 32). In this case, both key intermediates (3*S*,6*R*)-acetate and (3*R*,6*S*)-alcohol were obtained with high enantioselectivity (*E* > 195), c 50%. The enantiomerically pure (3*R*,6*S*)-alcohol was used as an intermediate in the synthesis of Stagonolide E. The chemoenzymatic synthesis of Pyrenophorol and Decarestrictine L involved the enantiomericaly pure allylic (3*S*,6*R*)-acetate as key intermediate (Scheme 32) [52].

### 3.22. Key Intermediates of Macrolide Antibiotic (−)-A26771B

**Scheme 33:**
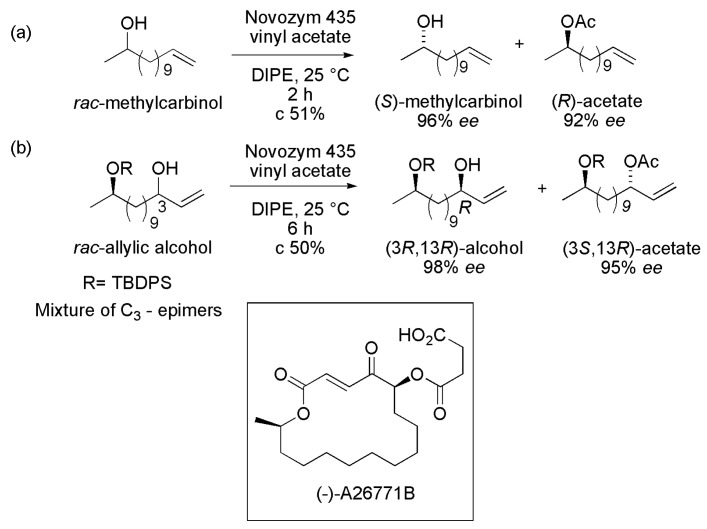
Kinetic enzymatic acylation of the (**a**) racemic tridec-12-en-2-ol and (**b**) allylic alcohol, affording key intermediates in the synthesis of the macrolide antibiotic (−)-A26771B [53].

The chemoenzymatic synthesis of the macrolide antibiotic (−)-A26771B involved the lipase-catalyzed acylation for resolving both a methylcarbinol and an allylic alcohol in order to establish the two stereogenic centers of the target molecule. A series of commercial lipases were investigated on the acetylation of the *rac*-methylcarbinol (tridec-12-en-2-ol) with vinyl acetate in hexane or DIPE at 25 °C. The best result was achieved when Novozym 435 (lipase B from *Candida antarctica* immobilized on acrylic resin) was used as the biocatalyst and DIPE as the solvent. This condition yielded (*S*)-methylcarbinol (96% *ee*) and the corresponding (*R*)-acetate (92% *ee*) after 2 h (c 51%), as seen in Scheme 33a. The same condition was used for the acetylation of the *rac*-allylic alcohol (Scheme 33b), which produced (3*R*,13*R*)-alcohol (98% *ee*) and (3*S*,13*R*)-acetate (95% *ee*) after 6 h (c 50%) [53].

### 3.23. Key Intermediate of Vitamin E Acetate

**Scheme 34:**
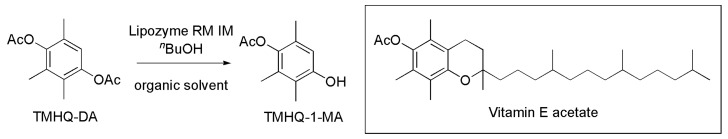
Lipase-catalyzed regioselective transesterification between TMHQ-DA and *n*-butanol on the preparation of TMHQ-1-MA, an intermediate in the synthesis of Vitamin E acetate [54].

Lipase-catalyzed regioselective transesterification between trimethylhydroquinone diacetate (TMHQ-DA) and *n*-butanol was applied on the preparation of the trimethylhydroquinone-1-monoacetate (TMHQ-1-MA) derivative, which is an important aromatic intermediate used in the synthesis of Vitamin E acetate (commercialized form of Vitamin E), as seen in Scheme 34. Seven commercially available lipases were screened, and the highest yield (99.1%) of the TMHQ-1-MA was obtained using Lipozyme RM IM (lipase from *Rhizomucor miehei*). No activity was observed when using lipase A (from *Aspergillus niger*) as an enzyme. Except for Lipozyme 435 (CAL-B, lipase B from *Candida antarctica* immobilized on macroporous polyacrylate resin), all active enzymes showed 100% regioselectivity on the formation of TMHQ-1-MA. Several reaction parameters were investigated and the optimum conditions for the Lipozyme RM IM catalyzed regioselective transesterification were: TMHQ-DA:*n*-butanol ratio of 1:1, 200 rpm, 50 °C, and TBME:*n*-hexane (3:7) as a solvent. In such conditions, the enzyme was active even after 20 cycles. A Ping-Pong bi-bi mechanism with *n*-butanol inhibition was proposed based on the initial rate data and concentration profiles [54].

### 3.24. Rasagiline Mesylate

**Scheme 35:**
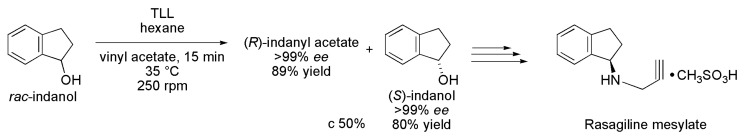
Enzymatic kinetic resolution *rac*-indanol in the chemoenzymatic synthesis of Rasagiline mesylate [55,56].

A straightforward chemoenzymatic synthesis of Rasagiline mesylate has been developed (Scheme 35). This drug is used in monotherapy of Parkinson patients at an early stage, and as an adjunct to moderate the advanced stage of the disease. The synthesis began with the chemical reduction of indanone to give *rac*-indanol in 86% yield. Then, *rac*-indanol was subjected to enzymatic kinetic resolution using the lipase from *Thermomyces lanuginosus* (TLL) immobilized on immobead-150 as a biocatalyst, hexane as an organic solvent, vinyl acetate as an acyl donor, at 35 °C and with 15 min of reaction time. In this case, (*R*)-indanyl acetate and (*S*)-indanol were obtained with >99% *ee*, c 50% and *E* > 200. Then, the (*S*)-indanol was subjected to a sequence of chemical steps, which included a Mitsunobu reaction, a Staudinger reaction, the introduction of the propargyl group and, finally, the reaction with methanesulfonic acid, which afforded Rasagiline mesylate. Immobilized lipase from *T. lanuginosus* was found to be an efficient biocatalyst to produce (*S*)-indanol with high enantioselectivity (>99% *ee*, *E* > 200) in hexane, at 35 °C and 15 min. Additionally, this enzyme was reused 10 times, maintaining both the activity and selectivity unchanged. This preparation of Rasagiline mesylate can be considered an environmentally sustainable strategy, since it involved the use of a biocatalyst that is commercially available, low-cost, stable, reusable for multiple reaction cycles and highly enantioselective [55,56].

**Scheme 36:**
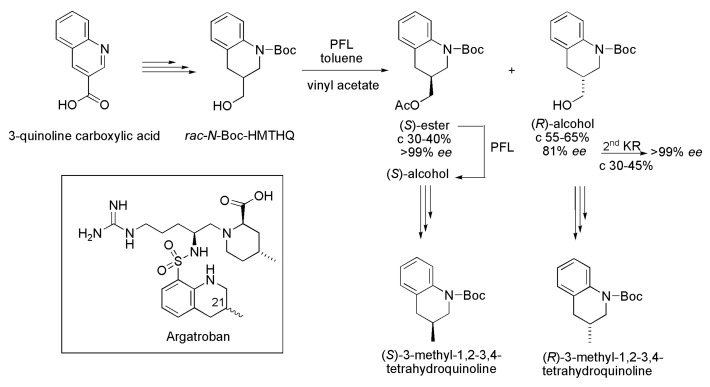
Enzymatic kinetic resolution of *rac*-*N*-Boc-HMTHQ to prepare the (*R*)- and (*S*)-3-methyl-1,2,3,4-tetrahydroquinoline, chiral intermediates for the preparation of the antithrombotic (21*R*)- and (21*S*)-Argatroban [57].

### 3.25. Argatroban

Argatroban is an inhibitor of thrombim, the protease that plays a key role in blood coagulation and fibrinolysis. The diastereoisomeric mixture of epimers ((21*R*)- and (21*S*)-epimers) is used as an antithrombotic drug, but the (21*S*)-isomer is twice as potent as 21*R*. The chiral intermediates (*R*)- and (*S*)-3-methyl-1,2,3,4-tetrahydroquinoline are used for the preparation of the antithrombotic (21*R*)- and (21*S*)-Argatroban. These intermediates were prepared using 3-quinoline carboxylic acid as a starting material. After some chemical steps, the *rac*-3-(1’-hydroxymethyl)-1-*tert*-butyloxycarbonyl-1,2,3,4-tetrahydroquinoline (*rac*-*N*-Boc-HMTHQ) was obtained, and subjected to kinetic resolution in the presence of lipase from *Pseudomonas fluorescens* (PFL), with toluene as a solvent and vinyl acetate as an acyl donor. After this procedure, the (*S*)-ester (>99% *ee* for c 30%–40%) and the (*R*)-alcohol (81% *ee* for c 55–65%) were obtained. For obtaining the enantiomerically enriched (*R*)-alcohol (>99% *ee*), a second kinetic resolution was performed under the aforementioned conditions using the (*R*)-alcohol (81% *ee*). In this case, the reaction was stopped at a conversion ranging from 30% to 45% (Scheme 36). The (*S*)-acetate (>99% *ee*) was converted into the corresponding (*S*)-alcohol by enzymatic hydrolysis in the presence of PFL. Subsequently, both (*S*)- and (*R*)-alcohol were subjected to a sequence of chemical steps which included tosylation, reaction with lithium aluminum hydride and removal of the Boc protecting group, leading to enantiomerically pure intermediates (*S*)- and (*R*)-3-methyl-1,2,3,4-tetrahydroquinoline [57].

### 3.26. Key Intermediate of the Paclitaxel Side Chain

**Scheme 37:**
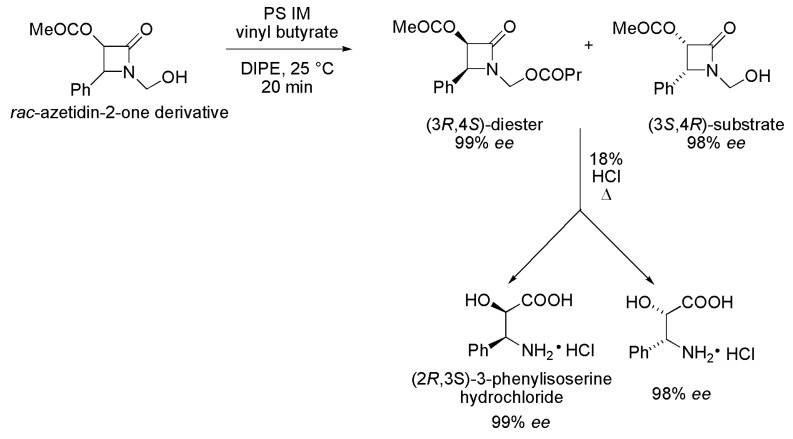
Enzymatic kinetic resolution of racemic *N*-hydroxymethylated *cis*-3-acetoxy-4-phenylazetidin-2-one (*rac*-azetidin-2-one derivative), affording the key intermediate for the Paclitaxel side chain, (2*R*,3*S*)-3-phenylisoserine [58].

The synthesis of a key intermediate for the Paclitaxel side chain, (2*R*,3*S*)-3-phenylisoserine, was performed by kinetic enzymatic resolution of racemic *N*-hydroxymethylated *cis*-3-acetoxy-4-phenylazetidin-2-one (*rac*-azetidin-2-one derivative), via acylation reaction (Scheme 37). Various parameters were evaluated such as lipases from *Burkholderia cepacia* (PS IM), *Candida antarctica* A (CAL-A), *Pseudomonas fluorescens* (lipase AK) and *C. rugosa* (lipase AY); acyl donors such as vinyl acetate, vinyl butyrate and 2,2,2-trifluoroethyl butyrate; temperature (4, 25 and 50 °C) and organic solvent (DIPE, toluene, TBME and 2-MeTHF). The best results were obtained in the presence of lipase PS IM, DIPE as a solvent, vinyl butyrate as an acyl donor, at 25 °C. The reaction was conducted with a higher amount of substrate (100 mg) and, after 20 min, the conversion reached 50%, with the formation of the diester (3*R*,4*S*) with >99% *ee* and the remaining substrate (3*S*,4*R*) with 98% *ee*, and *E* > 200, with traces of a byproduct from the migration of the acyl group. Both the (3*R*,4*S*)-diester and the remaining (3*S*,4*R*)-substrate were subjected to acid hydrolysis leading to (2*R*,3*S*)-3-phenylisoserine hydrochloride with >99% *ee* (key intermediate for the Paclitaxel side chain) and (2*S*,3*R*)-3-phenylisoserine hydrochloride with 98% *ee* [58].

### 3.27. Piperidine Derivative

**Scheme 38:**
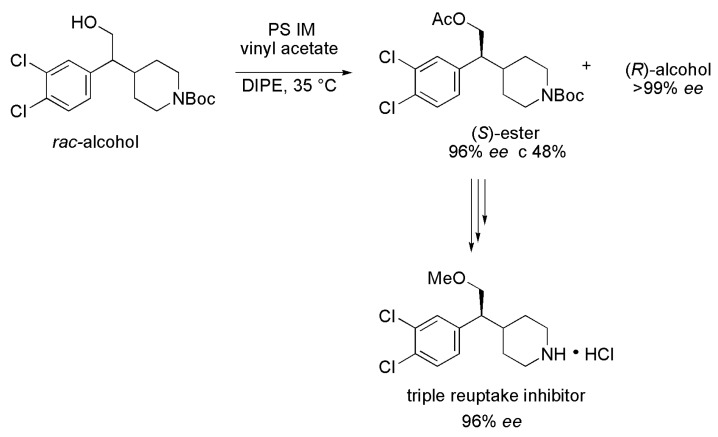
Enzymatic kinetic resolution of racemic *tert*-butyl 4-(1-(3,4-dichlorophenyl)-2-hydroxyethyl)piperidine-1-carboxylate (*rac*-alcohol), affording the novel triple reuptake inhibitor (*S*)-4-(1-(3,4-dichlorophenyl)-2-methoxyethyl)piperidine [59].

The chiral compound (*S*)-4-(1-(3,4-dichlorophenyl)-2-methoxyethyl)piperidine is a novel triple-reuptake inhibitor (inhibiting the reuptake of serotonin, norepinephrine and dopamine), which was created as a candidate for a novel antidepressant agent. Among the strategies to synthesize the aforementioned compound, it is possible to highlight the enzymatic kinetic resolution of racemic *tert*-butyl 4-(1-(3,4-dichlorophenyl)-2-hydroxyethyl)piperidine-1-carboxylate (*rac*-alcohol) via acetylation in the presence of lipases [59]. A preliminary enzyme screening was conducted using 38 lipases, wherein the lipase from *Pseudomonas* sp. immobilized on diatomite (Amano Enzyme Inc.) gave the best *E*-value (E 243). Several solvents were evaluated such as DIPE, THF, acetone, MeCN, DMF and TBME. In this case, DIPE showed the best performance leading to an *E*-value of 525. Thus, the kinetic resolution was performed in the optimized conditions using lipase PS IM (from *Pseudomonas* sp.), vinyl acetate as an acyl donor and DIPE as a solvent, at 35 °C. After the reaction reached 48% conversion, the product (*S*)-ester was obtained with 96% *ee* and (*R*)-alcohol with >99% *ee*, and *E* > 200 (Scheme 38). Then, after some chemical steps, the (*S*)-ester was converted into the triple-reuptake inhibitor (*S*)-4-(1-(3,4-dichlorophenyl)-2-methoxyethyl)piperidine with 96% *ee*. It is worth mentioning that the reaction was performed on a 10 g scale, and after 40 h it was necessary to add additional lipase and vinyl acetate. After over 24 h of agitation, the kinetic resolution gave an excellent enantioselectivity.

### 3.28. Tetrahydroquinolinol and Tetrahydrobenzoazepinol Derivatives

**Scheme 39:**
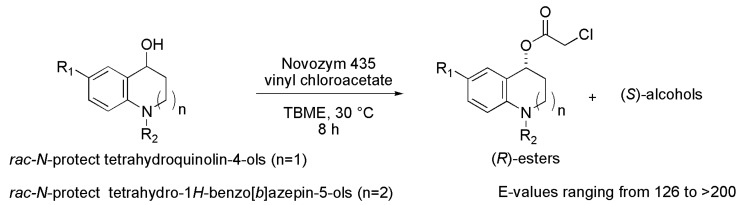
Enzymatic kinetic resolution of the racemic compounds *N*-protected tetrahydroquinolin-4-ols (*n* = 1) and *N*-protected tetrahydro-1*H*-benzo[*b*]azepin-5-ols (*n* = 2) to produce building blocks for drug candidates [60].

Tetrahydroquinolin-4-ols and tetrahydro-1*H*-benzo[*b*]azepin-5-ols, in chiral form, are building blocks for drug candidates due to their promising biological activities. A series of racemic *N*-protected tetrahydroquinolin-4-ols were chemically prepared from 1,2,3,4-tetrahydroquinoline. Subsequently, the *rac*-*N*-Boc-tetrahydroquinolin-4-ol (R_1_ = H; R_2_ = Boc) was subjected to kinetic resolution in the presence of lipases, using TBME as a solvent and vinyl acetate as an acyl donor, at 30 °C and for 8 h (*n* = 1, Scheme 39 and Table 4). Among the evaluated lipases (Amano lipase Type VII, CAL-A, Amano lipase A, Novozym 435, lipase RM IM and lipase TL IM), Novozym 435 lipase provided the highest enantioselectivity for the (*S*)-alcohol (98% *ee*) and the (*R*)-acetate (97% *ee*), c 50% and *E* > 200. Then, under the same conditions, various acyl donors were tested (vinyl chloroacetate, divinyl heptanedioate, vinyl hexanoate, vinyl undecanoate and vinyl 2,2-dimethylpropionate). In this case, vinyl chloroacetate was the most effective acyl donor, producing both (*R*)-ester and (*S*)-alcohol with >99% *ee*, c 50% and *E* > 200. Besides TBME, other solvents (DIPE, MeCN, toluene and *n*-hexane) were evaluated, but TBME proved to be more efficient in the kinetic resolution. After optimizing the reaction conditions (Novozym 435, TBME as a solvent, vinyl chloroacetate as an acyl donor, 30 °C, 8 h), the kinetic resolution was extended to the other racemic *N*-protected tetrahydroquinolin-4-ols containing substituent groups at the aromatic ring (C-6) and different *N*-protecting groups (Table 4). In general, the kinetic resolutions provided the (*R*)-esters ranging from 37% to 49% yields, 92% to >99% *ee*, and the (*S*)-alcohols ranging from 29% to 48% yields, 94% to >99% *ee*, with *E*-values ranging from 126 to >200. It is worth noting that the formation of any desired products was not observed when the substrate had the benzyl group linked to the nitrogen atom (*n* = 1; R_1_ = H; R_2_ = Bn), as seen in Scheme 39 and Table 4. In this same study, the authors conducted the kinetic resolution of racemic *N*-protected tetrahydro-1*H*-benzo[*b*]azepin-5-ols (R = Bz, 2-furoyl and Cbz), (*n* = 2, Scheme 39 and Table 4). As result, the kinetic resolutions produced the corresponding (*R*)-esters and (*S*)-alcohols in excellent yields (up to 44%) and enantioselectivity (*ee* up to 98% and *E* > 200) [60].

**Table 4:** Substrates used for the enzymatic kinetic resolution to produce tetrahydroquinolinol and tetrahydrobenzoazepinol derivatives [60].

Substrates	*n*	R_1_	R_2_
*rac*-*N*-protect tetrahydroquinolin-4-ols	1	H	Boc
		Cl	Boc
		Br	Boc
		OMe	Ac
		H	CO_2_Ph
		H	Cbz
*rac*-*N*-protect tetrahydro-1*H*-benzo[*b*]azepin-5-ols	2	H	Bz
		H	2-furoyl
		H	Cbz

### 3.29. Aminohydroxypiperidine Derivatives

**Scheme 40:**
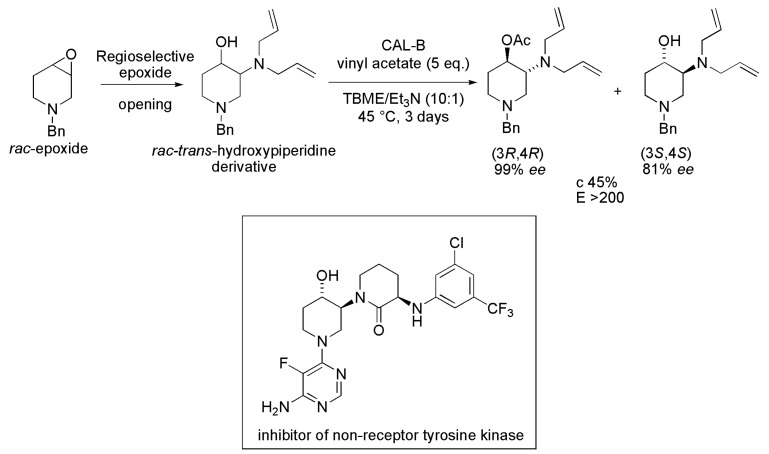
Enzymatic kinetic resolution of the racemic *trans*-hydroxypiperidine derivative to produce the orthogonally protected (3*R*,4*R*)-3-amino-4-hydroxypiperidine, which is present in the structure of an inhibitor of non-receptor tyrosine kinase [61].

The *trans*-3-amino-4-hydroxypiperidine moiety is present in the structures of some bioactive compounds, such as an inhibitor of non-receptor tyrosine kinase (Scheme 40) that is used for the treatment of auto-immune disorders. Enantiopure, orthogonally protected (3*R*,4*R*)-3-amino-4-hydroxypiperidine acetate was obtained by lipase-mediated kinetic acylation of the corresponding racemate. The racemic *trans*-*N*-benzyl-3-(diallylamino)-4-hydroxypiperidine (*trans*- hydroxypiperidine derivative) was obtained by the regioselective opening of the epoxide at the *rac*-1-benzyl-3,4-epoxypiperidine (*rac*-epoxide) with diallylamine. Later, it was subjected to the enzymatic transesterification reaction with vinyl acetate (5 eq.) as the acyl donor, lipases from *Candida antarctica* type A (lipase NZL-101) and *C. antarctica* B (Novozym 435), *Burkholderia cepacia* (PSL IM), *Pseudomonas fluorescens* (AK) and *Thermomyces lanuginosus* (TL IM), in TBME as the solvent, and at 30 °C. Although all lipases catalyzed the acylation of the substrate with the same stereochemical preference, (3*R*,4*R*)-product and (3*S*,4*S*)-substrate, the conversions were moderate (11%–31%). Thus, the influence of different reaction parameters (organic solvent, temperature, and triethylamine as additive) was studied in order to improve the reaction rate using the lipases CAL-A and CAL-B. The best result was obtained by using CAL-B, TBME/Et_3_N (10:1) as the solvent, three days of reaction time, and at 45 °C. In this case, the kinetic resolution yielded the (3*R*,4*R*)-product and (3*S*,4*S*)-substrate with enantiomeric excess of >99% and 81%, respectively, c 45% and *E* > 200 (Scheme 40). It should be mentioned that when the reaction was performed without triethylamine as an additive, a significant decrease in the reaction rate was observed (from three to seven days) [61].

### 3.30. Azole Derivatives

**Scheme 41:**
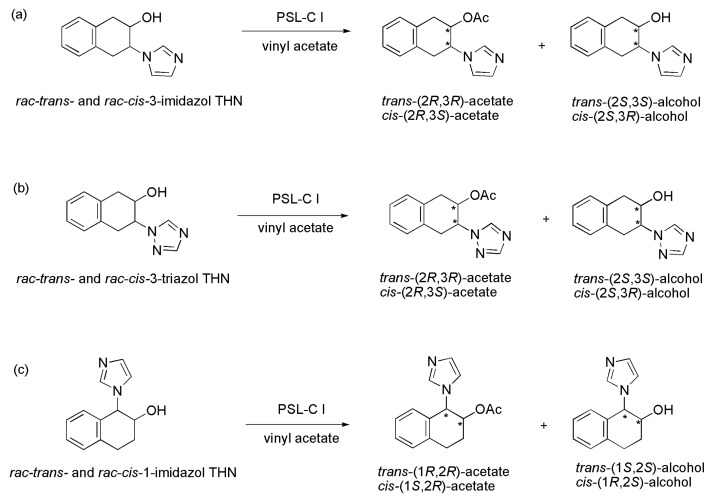
Enzymatic kinetic resolution of the racemic *trans*- and *cis*-3-imidazol THN (**a**); racemic *trans*- and *cis*-3-triazol THN (**b**) and racemic *trans*- and *cis*-1-imidazol THN (**c**) [62].

The azole subunit is part of the structure of a number of drugs such as Econazole, Fluconazole, Ketoconalole, Miconazole, Voriconazole and Ravuconazole. It is noteworthy that cycloalkyl azoles have been described as potent antileishmanial agents. A new family of racemic *trans*- and *cis*-azole derivatives was prepared and submitted to a kinetic resolution through transesterification in the presence of lipases (Scheme 41). The kinetic resolution was conducted with the racemic *trans*-3-(1*H*-imidazol-1-yl)-1,2,3,4-tetrahydronaphthalen-2-ol (*rac*-*trans*-3-imidazol THN) in the presence of vinyl acetate as the acyl donor and THF as the solvent (Scheme 41a and Table 5). Several lipases were evaluated, such as lipases from *Candida antarctica* B (CAL-B, immobilized by adsorption in Lewatit E), from *C. antarctica* A (CAL-A), from *C. rugosa* (CRL), lipase AK from *Pseudomonas fluorescens*, from *P. cepacia* (PSL), from *Rhizomucor miehei* (RML), porcine pancreatic lipase (PPL), and from *Thermomyces lanuginosus* (TLL). Among them, only PSL produced an appreciable degree of activity with good selectivity. Subsequently, several commercially available types of PSL were evaluated, and PSL-C I (immobilized onto a ceramic carrier) was the most efficient (with a ratio of 1:1 lipase/substrate in weight), producing (after 48 h at 30 °C) *trans*-(2*R*,3*R*)-acetate in 95% *ee* and *trans*-(2*S*,3*S*)-alcohol with 83% *ee*, c 46%, *E*-value of 102. PSL-C I was also the most efficient in the kinetic resolution of the *cis*-isomer. After 24 h at 45 °C, using the ratio 2:1 of lipase:substrate in weight, the *cis*-(2*R*,3*S*)-acetate (95% *ee*) and *cis*-(2*S*,3*R*)-alcohol (71% *ee*) were obtained with c 43% and an E-value of 83 (Scheme 41a and Table 5). The strategy used to prepare the chiral imidazole derivatives was used to enantioselectively obtain the *trans*- and *cis*-triazol compounds (Scheme 41b and Table 5). Again, PSL-C I was the most efficient enzyme to promote the kinetic resolution of both racemic *trans*- and *cis*-3-(1*H*-1,2,4-triazol-1-yl)-1,2,3,4-tetrahydronaphthalen-2-ols (*rac*-*trans*- and *rac*-*cis*-3-triazol THN). The experiments were performed using THF as the solvent and vinyl acetate as the acyl donor, at 30 °C and 48 h. The reaction with the *rac*-*trans*- isomer provided the *trans*-(2*R*,3*R*)-acetate with 99% *ee* and *trans*-(2*S*,3*S*)-alcohol with 93% *ee*, c 48% and *E* > 200. In the case of the *rac*-*cis*-isomer, it was obtained from the *cis*-(2*R*,3*S*)-acetate with >99% *ee* and *cis*-(2*S*,3*R*)-alcohol with 29% *ee*, c 23% and *E* > 200 (Scheme 41b and Table 5) [62]. Tetrahydronaphthylazoles are known to exhibit antileishmanial properties, which has inspired the authors [62] to resolve the racemate of *trans*- and *cis*-1-(1*H*-imidazol-1-yl)-1,2,3,4-tetrahydronaphthalen-2-ols (*rac*-*trans*- and *rac*-*cis*-1-imidazol THN) using the same aforementioned enzymatic methodology. The kinetic resolutions of these isomers were conducted in THF as a solvent and vinyl acetate as an acyl donor, at 30 °C and for 48 h. The lipases CAL-B and PSL-C I were tested and the latter led to the best results. Thus, for the *rac*-*trans*-isomer, both the *trans*-(1*R*,2*R*)-acetate in 96% *ee* and *trans*-(1*S*,2*S*)-alcohol with 85% *ee* (c 43% and an *E*-value of 133) were obtained. In the case of the *rac*-*cis*-isomer, the compounds *cis*-(1*S*,2*R*)-acetate and *cis*-(1*R*,2*S*)-alcohol with 98% *ee* (c 50% and *E* > 200) were obtained (Scheme 41c and Table 5).

**Table 5:** Results from the enzymatic kinetic resolution of the racemic *trans*- and *cis*-3-imidazol THN (a), racemic *trans*- and *cis*-3-triazol THN (b) and racemic *trans*- and *cis*-1-imidazol THN (c) [62].

Scheme 41	Substrate	c (%)	*ee*_P_ (%) Acetate	*ee*_S_ (%) Alcohol	*E*
a	*trans*-3-imidazol THN	46	95	83	102
	*rac*-*cis*-3-imidazol THN	43	95	71	83
b	*rac*-*trans*-3-triazol THN	48	99	93	>200
	*rac*-*cis*-3-triazol THN	23	>99	29	>200
c	*rac*-*trans*-1-imidazol THN	43	96	85	133
	*rac*-*cis*-1-imidazol THN	50	98	98	>200

### 3.31. Benzoin Derivative

**Scheme 42:**
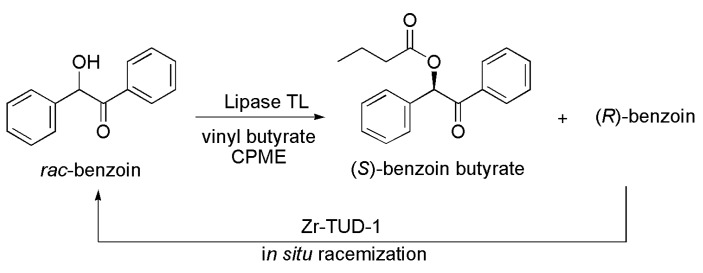
Dynamic enzymatic kinetic resolution (DKR) of *rac*-benzoin in conventional batch or continuous flow [63].

Chiral α-hydroxy ketones are important building blocks in the syntheses of several biologically active compounds such as pharmaceuticals, agrochemicals and pheromones. Aiming to obtain the (*S*)-benzoin butyrate, a study for the dynamic enzymatic kinetic resolution (DKR) of racemic benzoin via acylation was developed [63]; an efficient DKR is needed for competing with other biocatalytical approaches, such as the enantioselective reduction of benzyl ketones, which has been reported as a useful technique for preparing (*S*)-benzoins [64,65]. The reactions were conducted in batch or continuous mode using lipase from *Pseudomonas stutzeri* (lipase TL) immobilized on Accurel carrier MP1001 [63]. Various solvents were evaluated in relation to TL lipase activity such as toluene, 2-MeTHF, 1,3-dioxolane, cyclopentyl methyl ether (CPME), as well as some solvents classified as deep eutectic solvents (DESs). At the same time, a study was conducted to verify the influence of the amount of water in the solvents on the enzyme activity. As result, dry CPME was the most efficient solvent for the catalytic activity of the lipase TL as well as for catalyst activity of Zr-TUD-1 (a three-dimensional mesoporous silicate containing zirconium), with this latter used for *in situ* racemization of the (*R*)-benzoin. The DKR of *rac*-benzoin, in batch mode, was carried out at 50 °C in the presence of vinyl butyrate (6 eq.) as the acyl donor, dried CPME as the solvent, Zr-TUD-1 as the catalyst and immobilized TL lipase as the enzyme. After 5 h, (*S*)-benzoin butyrate (c 98.2% and 99% *ee*) was obtained. Subsequently, the DKR of *rac*-benzoin was investigated in a continuous flow system in the presence of vinyl butyrate (3 eq.), TL lipase, and Zr-TUD-1 in CPME. After 25 h, the (*S*)-benzoin butyrate was obtained with a maximum conversion of 40%, decreasing to 11% at 76 h. The enantiomeric excess values (*ee*) of the (*S*)-benzoin butyrate were ≥98% throughout the process (Scheme 42) [63].

The DKR in the presence of lipase is an important approach for obtaining enantiomerically pure intermediates for the synthesis of biologically active compounds with both *ee* and conversion up to 100%. Recently, an elegant review covering this subject was published [66].

## 4. Complementary Approaches: Hydrolysis and Esterification

### 4.1. Acetonide Derivatives

**Scheme 43:**
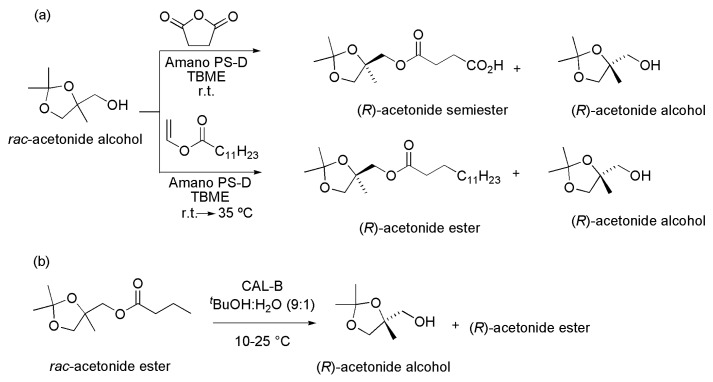
Syntheses of (*R*)-acetonide derivatives by enantiocomplementary kinetic resolution via acylation of the racemic *rac*-acetonide alcohol (**a**) and via hydrolysis of (*R*)-acetonide ester (**b**) [67].

The compound ((*R*)-2,2,4-trimethyl-1,3-dioxolan-4-yl)-methanol ((*R*)-acetonide alcohol), a chiral intermediate used in the synthesis of some bioactive compounds, was prepared in high *ee* by the enzymatic resolution of its racemate form (Scheme 43a). In this case, both succinic anhydride and vinyl laurate were used as acyl donors, and Amano PS-D lipase was the selected enzyme. The (*S*)-acetonide alcohol was selectively acylated (with TBME as the solvent) and yielded the (*R*)-acetonide semiester (with succinic anhydride as the acyl donor) and (*R*)-acetonide ester (with vinyl laurate as the acyl donor), besides the remaining (*R*)-acetonide alcohol (Scheme 43a). The (*R*)-acetonide alcohol was also obtained by kinetic resolution of the corresponding racemic ester via hydrolysis using CAL-B (Novozym 435) as the lipase, and *t*-BuOH:H_2_O (9:1) as the solvent (Scheme 43b) [67].

### 4.2. Vitamin D Analogues

**Scheme 44:**
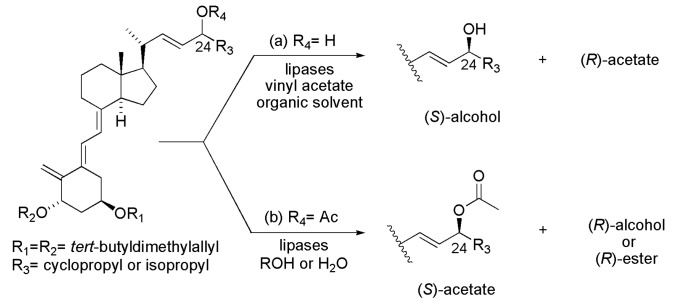
Synthesis of vitamin D analogues by enzymatic kinetic resolution via esterification (**a**) or (**b**) solvolysis of the corresponding esters [68].

Vitamin D plays a vital role in maintaining calcium levels within the normal range. Analogues of vitamin D have been prepared in order to obtain compounds with calcemic activity. Such analogues were obtained by separation of a mixture of epimers (at C-24 containing an OH group) using two approaches, (a) enzymatic kinetic resolution via esterification or (b) solvolysis of the corresponding esters, and the epimer of interest (with biological activity) was the one with *S* configuration. In the first approach (route a), the esterifications were carried out using an acylating agent (especially vinyl acetate), organic solvent (especially hexane or DIPE) and lipases from *Alcalagenes* sp. or *Pseudomonas* sp. (in free or immobilized form). The reactions were monitored by HPLC to ensure diastereomeric excess amounts in the range of 80%–95%. In this case, the desired epimer is the remaining alcohol (*S* configuration at C-24), as seen in Scheme 44a. In the second approach (route b), esterified vitamin D analogues have undergone alcoholysis or hydrolysis in the presence of the same lipases used in route a. These reactions gave a diastereomeric excess in the range of 80%–95% (Scheme 44b) and the desired epimer was the non-hydrolyzed (*S*)-acetate [68].

### 4.3. Arylalkylcarbinol Derivatives

**Scheme 45:**
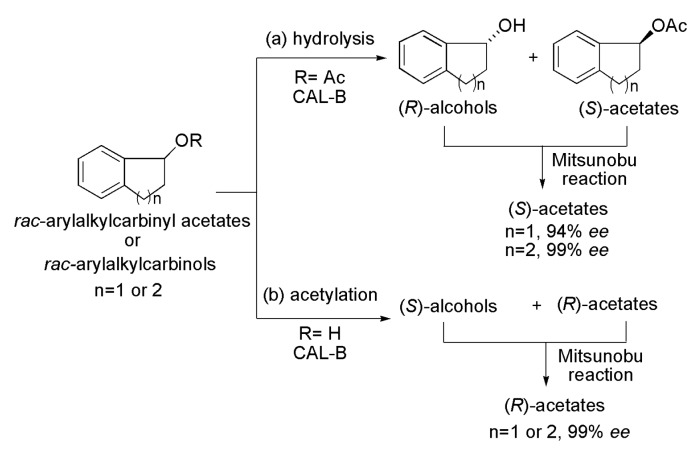
Complementary enzymatic kinetic resolution of *rac*-arylalkylcarbinyl acetates or *rac*-arylalkylcarbinols combined with Mitsunobu reaction on the preparation of (*S*)- and (*R*)-arylalkylcarbinyl acetates via hydrolysis (**a**) or acylation (**b**), respectively [69].

*Candida antarctica* lipase B (CAL-B, Novozym 435) was investigated as a biocatalyst in the complementary preparation of (*S*)- and (*R*)-arylalkylcarbinol acetates, which are important intermediates for the syntheses of several compounds with industrial application. This enzyme was used in the hydrolysis of racemic arylalkylcarbinyl acetates (route a) and produced both (*R*)-alcohols and (*S*)-acetates in high enantioselectivities. Then, the mixture of these compounds was subjected to Mitsunobu reaction to yield the (*S*)-acetates as the only products (94% *ee* (*n* = 1) and 99% *ee* (*n* = 2)), as seen in Scheme 45a. The complementary process was conducted using CAL-B on the catalyzed acylation of the *rac*-arylalkylcarbinols, producing the (*S*)-alcohols and (*R*)-acetates in high enantioselectivities (route b). Subsequently, these mixtures were submitted to a Mitsunobu reaction and provided only the (*R*)-acetates (99% *ee* (*n* = 1 or 2)), as seen in Scheme 45b [69].

### 4.4. Key Intermediate of Levofloxacin

**Scheme 46:**
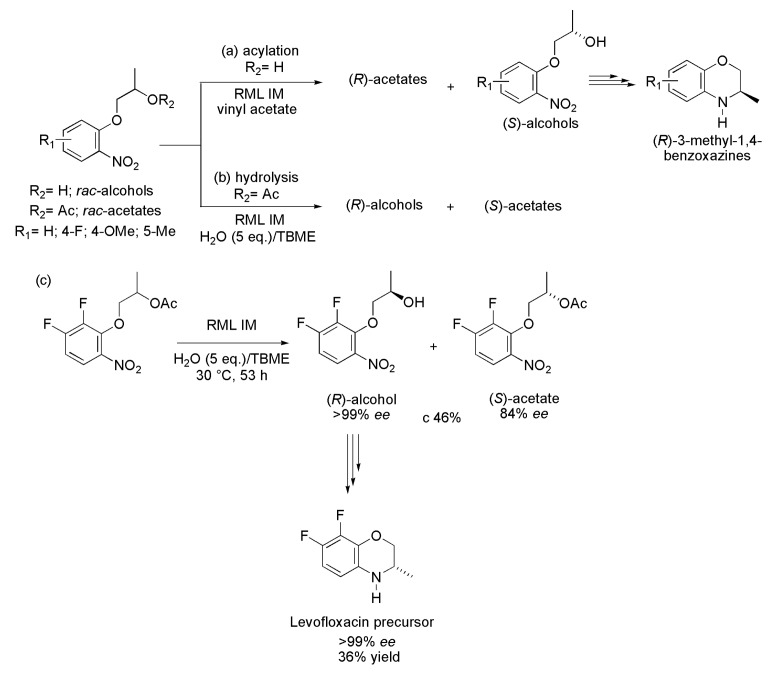
Enzymatic kinetic resolution of racemic alcohols 1-(2-nitrophenoxy)propan-2-ols via acetylation (**a**) or via hydrolysis (**b**) of the corresponding racemic acetates. Production of (*S*)-7,8-difluoro-3-methyl-3,4-dihydro-2*H*-benzo[*b*][1,4]oxazine precursor of the antibacterial drug Levofloxacin (**c**) [70].

The benzoxazine moiety is part of the structures of a series of molecules with biological activities such as antibacterial, anticancer, antifungal and antimicrobial. A number of 1,4-benzoxazine derivatives (with or without substituents at the aromatic ring) were prepared by a combination of chemical steps and lipase-mediated kinetic resolution [70]. The enzymatic step was conducted with *rac*-1-(2-nitrophenoxy)propan-2-ols (*rac*-alcohols), via acylation (route a), and with the corresponding *rac*-acetates, via hydrolysis (route b), as seen in Scheme 46. These two approaches are considered complementary lipase-catalyzed processes. The process of kinetic resolution via acetylation was first evaluated using the *rac*-1-(2-nitrophenoxy)propan-2-ol as a substrate, in the presence of vinyl acetate as an acyl donor, TBME as a solvent, at 30 °C and with 5 h of reaction time. After screening lipases from *Candida antarctica* (CAL-A), from *Pseudomonas cepacia* (PSL C-I), from *C. antarctica* B (CAL-B) and from *Rhizomucor miehei* in immobilized form (RML IM), it was found that the latter (RM IM) was the most efficient. In the aforementioned conditions, the (*R*)-acetate (94% *ee*) and the remaining (*S*)-alcohol (89% *ee*) were obtained with c 48%. The study was extended to the *rac*-alcohols with substituents at the aromatic ring (4-F, 4-OMe and 5-Me), leading to the (*R*)-acetates (93%–95% *ee*) and the remaining (*S*)-alcohols (89%–94% *ee*) with c 48%–50% and *E*-values ranging from 103 to >200 (Scheme 46a and Table 6). By a sequence of reactions, the (*R*)-3-methyl-3,4-dihydro-2*H*-benzo[*b*][1,4]oxazine derivatives ((*R*)-3-methyl-1,4-benzoxazines) were synthesized from the (*S*)-alcohols. The second approach (route b) was related to the resolution of the corresponding *rac*-acetates via hydrolysis reaction. In this case, the reactions were carried out in the presence of RML IM, at 30 °C, using TBME and water (5 eq.) as solvents. After 52 h, the (*R*)-alcohols (96% to >99% *ee*) and the remaining (*S*)-acetates (91%–97% *ee*) were obtained with c 48%–50% and *E* > 200 (Scheme 46b and Table 6). Due to the successful strategies for obtaining the chiral 1,4-benzoxazines, the authors performed the production of the (*S*)-7,8-difluoro-3-methyl-3,4-dihydro-2*H*-benzo[*b*][1,4]oxazine, precursor of the potent antibacterial Levofloxacin (Scheme 46c). Then, the kinetic resolution of the *rac*-1-(2,3-difluoro-6-nitrophenoxy)propan-2-yl acetate was performed, via hydrolysis in the presence of lipase RM IM, leading to the (*R*)-alcohol (>99% *ee*) and the remaining (*S*)-acetate (84% *ee*) with c 46%. Subsequently, after several chemical steps, the (*R*)-alcohol was converted into the (*S*)-7,8-difluoro-3-methyl-3,4-dihydro-2*H*-benzo[*b*][1,4]oxazine (36% yield, >99% *ee*) Levofloxacin precursor [70].

**Table 6:** Results from the enzymatic kinetic resolution of racemic alcohols 1-(2-nitrophenoxy)propan-2-ols via acetylation (a) or via hydrolysis (b) of the corresponding racemic acetates [70].

Scheme 46	Acetates		Alcohols		c (%)	*E*
	*ee* (%)	Yield (%)	*ee* (%)	Yield (%)		
a	93–95	45–47	89–94	44–48	48–50	103–>200
b	91–97	41–48	96–>99	44–47	48–50	>200

## 5. Conclusions

Recent examples of the use of lipases in the preparation of enantiomerically pure active pharmaceutical ingredients (APIs) and their intermediates were reviewed, confirming the importance of these enzymes in obtaining compounds with high added value. Most reported biocatalytic processes refer to kinetic resolutions of racemic substrates, which occur under mild conditions with a high degree of regio- or enantioselectivity. Among the examples presented, the action of lipases can be seen in a wide range of substrates with varied structures such as aromatic, heteroaromatic (containing atoms of nitrogen, sulfur, chlorine, *etc.*), as well as aliphatic with either open or cyclic chain (branched or not). The substances produced by the action of lipases, in the examples herein, are pharmaceutical intermediates or drugs, which makes the biocatalytic process of great interest in the pharmaceutical industry. Additionally, it was also possible to confirm the versatility of lipases for acting in either aqueous medium or organic solvents for the hydrolysis of esters, the acylation of alcohols or the esterification of carboxylic acid, allowing the production of the desired stereoisomer. It highlights the operational simplicity, as it is not necessary to add cofactors in the reaction system, and the ease of finding a wide range of commercially available and relatively low-cost lipases. The use of immobilized lipase often allows the reuse of the enzymatic system for several cycles, making the process more effective and economically viable. In summary, it is clear that lipases will continue to be an excellent alternative for obtaining biologically active compounds.

## References

[1] Gotor-Fernandez V., Brieva R., Gotor V. (2006). Lipases: Useful biocatalysts for the preparation of pharmaceuticals. J. Mol. Catal. B..

[2] Tao J., Zhao L., Ran N. (2007). Recent advances in developing chemoenzymatic processes for active pharmaceutical ingredients. Org. Process Res. Dev..

[3] Pollard D.J., Woodley J.M. (2007). Biocatalysis for pharmaceutical intermediates: The future is now. Trends Biotechnol..

[4] Patel R.N. (2008). Synthesis of chiral pharmaceutical intermediates by Biocatalysis. Coord. Chem. Rev..

[5] Tao J., Xu J.-H. (2009). Biocatalysis in development of green pharmaceutical processes. Curr. Opin. Chem. Biol..

[6] Patel R.N. (2011). Biocatalysis: Synthesis of key intermediates for development of pharmaceuticals. ACS Catal..

[7] Anand N., Kapoor M., Ahmad K., Koul S., Parshad R., Manhas K.S., Sharma L.R., Qazi G.N., Taneja S.C. (2007). *Arthrobacter* sp.: A lipase of choice for the kinetic resolution of racemic arylazetidinone precursors of taxanoid side chains. Tetrahedron.

[8] Rimoldi M., Pellizzoni G., Facchetti F.E., Molinari F., Zerla D.S., Gandolfi R. (2011). Chemo- and biocatalytic strategies to obtain phenylisoserine, lateral chain of taxol by asymmetric reduction. Tetrahedron.

[9] Forró E., Fülöp F. (2010). A new enzymatic strategy for the preparation of (2*R*,3*S*)-3-phenylisoserine: A key intermediate for the Taxol side chain. Tetrahedron.

[10] Forró E., Fülöp F. (2010). New enzymatic two-step cascade reaction for the preparation of a key intermediate for the taxol side-chain. Eur. J. Org. Chem..

[11] Cui J.J., Tran-Dubé M., Shen H., Nambu M., Kung P.-P., Pairish M., Jia L., Meng J., Funk L., Botrous I. (2011). Structure based drug design of crizotinib (PF-02341066), a potent and selective dual inhibitor of mesenchymal-epithelial transition factor (c-MET) kinase and anaplastic lymphoma kinase (ALK). J. Med. Chem..

[12] Kung P.-P., Martinez C., Tao J. (2008). Enantioselective Biotransformation for Preparation of Protein Tyrosine Kinase Inhibitor Intermediates.

[13] Felluga F., Pitacco G., Valentin E., Venneri C.D. (2008). A facile chemoenzymatic approach to chiral non-racemic β-alkyl-γ-amino acids and 2-alkylsuccinic acids. A concise synthesis of (*S*)-(+)-Pregabalin. Tetrahedron.

[14] Li X.-J., Zheng R.-C., Ma H.-Y., Zheng Y.-G. (2014). Engineering of *Thermomyces lanuginosus* lipase Lip: Creation of novel biocatalyst for efficient biosynthesis of chiral intermediate of Pregablin. Appl. Microbiol. Biotechnol..

[15] Rouf A., Gupta P., Aga M.A., Kumar B., Chaubey A., Parshad R., Taneja S.C. (2012). Chemoenzymatic synthesis of piperoxan, prosympal, dibozane, and doxazosin. Tetrahedron.

[16] Singh A., Goel Y., Rai A.K., Banerjee U.C. (2013). Lipase catalyzed kinetic resolution for the production of (*S*)-3-[5-(4-fluoro-phenyl)-5-hydroxy-pentanoyl]-4-phenyl-oxazolidin-2-one: An intermediate for the synthesis of ezetimibe. J. Mol. Catal. B.

[17] Goswami A., Guo Z., Qiu Y. (2014). Process for Resolving cyclopropyl diesters.

[18] Takaç S., Bakkal M. (2007). Impressive effect of immobilization conditions on the catalytic activity and enantioselectivity of *Candida rugosa* lipase toward *S*-Naproxen production. Pro. Biochem..

[19] Sayin S., Akoz E., Yilmaz M. (2014). Enhanced catalysis and enantioselective resolution of racemic naproxen methyl ester by lipase encapsulated within iron oxide nanoparticles coated with calix[8]arene valeric acid complexes. Org. Biomol. Chem..

[20] Sharifabad M.E., Hodgson B., Jellite M., Mercer T., Sen T. (2014). Enzyme immobilised novel core–shell superparamagnetic nanocomposites for enantioselective formation of 4-(*R*)-hydroxycyclopent-2-en-1-(*S*)-acetate. Chem. Commun..

[21] Hu C., Wang N., Zhang W., Zhang S., Meng Y., Yu X. (2015). Immobilization of *Aspergillus terreus* lipase in self-assembled hollow nanospheres for enantioselective hydrolysis of ketoprofen vinyl ester. J. Biotechnol..

[22] Zhang W.-W., Jia J.-Q., Wang N., Hu C.-L., Yang S.-Y., Yu X.-Q. (2015). Improved activity of lipase immobilized in microemulsion-based organogels for (*R*,*S*)-ketoprofen ester resolution: Long-term stability and reusability. Biotechnol. Rep..

[23] Reis P., Holmberg K., Watzke H., Leser M.E., Miller R. (2009). Lipases at interfaces: A review. Adv. Colloid Interface Sci..

[24] Torres S.Y., Verdecia Y., Rebolledo F. (2015). Chemoenzymatic approach to optically active 1,4-dihydropyridine derivatives. Tetrahedron.

[25] Gu J., Rupeen M.E., Raveendranath P., Chew W., Shaw C.C. (2007). Proline cci-779 (Proline-Rapamycin 42-Ester with 2,2-Bis(hydroxymethyl) Propionic Acid) and Two-Step Enzymatic Synthesis of Proline cci-779 and cci-779 Using Microbial Lipase.

[26] Ferraboschi P., Colombo D., de Mieri M., Grisenti P. (2009). First chemoenzymatic synthesis of immunomodulating macrolactam pimecrolimus. Tetrahedron Lett..

[27] Grisenti P., Reza E.S., Verza E. (2015). A Chemo-Enzymatic Approach to the Synthesis of Primecrolimus.

[28] Bhuniya R., Nanda S. (2011). Asymmetric synthesis of the active form of loxoprofen and its analogue. Tetrahedron.

[29] Spizzo P., Basso A., Ebert C., Gardossi L., Ferrario V., Romano D., Molinari F. (2007). Resolution of (*R*,*S*)-flurbiprofen catalysed by dry mycelia in organic solvente. Tetrahedron.

[30] Tamborini L., Romano D., Pinto A., Bertolani A., Molinari F., Conti P. (2012). An efficient method for the lipase-catalysed resolution and in-line purification of racemic flurbiprofen in a continuous-flow reactor. J. Mol. Catal. B.

[31] Tamborini L., Romano D., Pinto A., Contente M., Iannuzzi M.C., Conti P., Molinari F. (2013). Biotransformation with whole microbial systems in a continuous flow reactor: Resolution of (*RS*)-flurbiprofen using *Aspergillus oryzae* by direct esterification with ethanol in organic solvent. Tetrahedron Lett..

[32] Pastre J.C., Browne D.L., Ley S.V. (2013). Flow chemistry syntheses of natural products. Chem. Soc. Rev..

[33] Itabaiana I., Miranda L.S.M., Souza R.O.M.A. (2013). Towards a continuous flow environment for lipase-catalyzed reactions. J. Mol. Catal. B.

[34] Marszałł M.P., Siódmiak T. (2012). Immobilization of *Candida rugosa* lipase onto magnetic beads for kinetic resolution of (*R*,*S*)-ibuprofen. Catal. Commun..

[35] Shinde S.D., Yadav G.D. (2015). Insight into microwave assisted immobilized *Candida Antarctica* lipase B catalyzed kinetic resolution of *R*,*S*-(±)-ketorolac. Process Biochem..

[36] Bizerra A.M.C., Montenegro T.G.C., Lemos T.L.G., de Oliveira M.C.F., de Mattos M.C., Lavandera I., Gotor-Fernandez V., Gonzalo G., Gotor V. (2011). Enzymatic regioselective production of chloramphenicol esters. Tetrahedron.

[37] Da Silva M.R., Montenegro T.G.C., de Mattos M.C., de Oliveira M.C.F., de Lemos T.L.G., de Gonzalo G., Lavandera I., Gotor-Fernandez V., Gotor V. (2014). Regioselective preparation of thiamphenicol esters through lipase-catalyzed processes. J. Braz. Chem. Soc..

[38] Borowiecki P., Balter S., Justyniak I., Ochal Z. (2013). First chemoenzymatic synthesis of (*R*)- and (*S*)-1-(9*H*-fluoren-9-yl)ethanol. Tetrahedron.

[39] Stürmer R. (2008). Method for producing enantiomer-pure aminoalcohols.

[40] Yamashita S., Mase N., Takabe K. (2008). Chemoenzymatic total synthesis and determination of the absolute configuration of (*S*)-nebracetam. Tetrahedron.

[41] Araujo D.M.F., Vieira G.A.B., Mattos M.C., Lemos T.L.G., Oliveira M.C.F., Melo V.M.M., Gonzalo G., Gotor-Fernandez V., Gotor V. (2009). Chemoenzymatic preparation of a biologically active naphthoquinone from *Tabebuia impetiginosa* using lipases or alcohol dehydrogenases. J. Mol. Catal. B.

[42] Kamal A., Khanna G., Krishnaji T., Ramu R. (2010). Chemoenzymatic Process for the Stereoselective Preparation of (*R*)-γ-Amino-β-hydroxybutyric Acid or (*R*)-Carnitine from 3,4-Dihydroxybutanenitrile.

[43] Nagarapu L., Gaikwad H.K., Bantu R., Manikonda S.R. (2011). Chemoenzymatic synthesis with lipase catalyzed resolution and evaluation of antitumor activity of (*R*/*S*)-2-[2-hydroxy-3-(4-phenylpiperazin-1-yl)propyl]-1*H*-pyrrolo[3,4-*b*]quinolin-3(2*H*)-one. Eur. J. Med. Chem..

[44] Yadav G.D., Devendran S. (2012). Lipase catalyzed kinetic resolution of (±)-1-(1-naphthyl) ethanol under microwave irradiation. J. Mol. Catal. B.

[45] Wamvakides A., Moutsos V., Schmitt M. (2013). Synthesis of (+) and (ࢤ) 1-(5,5-Diphenyltetrahydrofuran-3-yl)-*N*,*N*-dimethylmethanamine, (+) and (ࢤ) 1-(2,2-Diphenyltetrahydrofuran-3-yl)-*N*,*N*-dimethylmethanamine and (+) and (ࢤ) 1-(2,2-Diphenyltetrahydrofuran-3-yl)-*N*-methylmethanamine.

[46] González-Sabín J., Ríos-Lombardía N., Gotor V., Morís F. (2013). Enzymatic transesterification of pharmacologically interesting β-aminocycloalkanol precursors. Tetrahedron.

[47] Jacobsen E.E., Lie A., Frigstad M.M.H., el-Behairy M.F., Ljones T., Wohlgemuth R., Anthonsen T. (2013). Desymmetrization of cbz-serinol catalyzed by crude pig pancreatic lipase reveals action of lipases with opposite enantioselectivity. J. Mol. Catal. B.

[48] Da Silva M.R., de Mattos M.C., de Oliveira M.C.F., de Lemos T.L.G., Ricardo N.M.P.S., de Gonzalo G., Lavandera I., Gotor-Fernández V., Gotor V. (2014). Asymmetric chemoenzymatic synthesis of *N*-acetyl-α-amino esters based on lipase-catalyzed kinetic resolutions through interesterification reactions. Tetrahedron.

[49] Devendran S., Yadav G.D. (2014). Lipase-catalyzed kinetic resolution of (±)-1-(2-furyl) ethanol in nonaqueous media. Chirality.

[50] Karmee S.K. (2011). The synthesis, properties, and applications of ascorbyl esters. Lipid. Technol..

[51] Kharrat N., Aissa I., Sghaier M., Bouaziz M., Sellami M., Laouini D., Gargouri Y. (2014). Lipophilization of ascorbic acid: A monolayer study and biological and antileishmanial activities. J. Agric. Food Chem..

[52] Chatterjee S., Ghadigaonkar S., Sur P., Sharma A., Chattopadhyay S. (2014). A chemoenzymatic synthesis of hept-6-ene-2,5-diol stereomers: Application to asymmetric synthesis of decarestrictine L, pyrenophorol, and stagonolide E. J. Org. Chem..

[53] Chatterjee S., Sharma A., Chattopadhyay S. (2014). Chemoenzymatic synthesis of macrolide antibiotic (−)-A26771B. RSC Adv..

[54] Wei C., Fu X.-F., Wang Z., Yu X.-J., Zhang Y.-J., Zheng J.-Y. (2014). Efficient synthesis of vitamin E intermediate by lipase-catalyzed regioselective transesterification. J. Mol. Catal. B.

[55] Fonseca T.S., Silva M.R., Oliveira M.C.F., Lemos T.L.G., Marques R.A., Mattos M.C. (2015). Chemoenzymatic synthesis of rasagiline mesylate using lipases. Appl. Catal. A.

[56] Carvalho A.C.L.M., Araujo D.M.F., Gonçalves L.R.B., Mattos M.C., Silva M.R., Oliveira M.C.F., Marques R.A., Lemos T.L.G., Fonseca T.S., Oliveira U.M.F. (2015). Desenvolvimento de um Processo Biocatalítico para a Produção do (*S*)-Indanol, Precursor do Fármaco Mesilato de Rasagilina.

[57] Grisenti P. (2015). Biocatalyzed Synthesis of the Optically Pure (*R*) and (*S*) 3-Methyl-1,2,3,4-tetrahydroquinoline and Their Use as Chiral Synthons for the Preparation of the Antithrombotic (21*R*)- and (21*S*)-Argatroban.

[58] Forró E., Galla Z., Nádasdia Z., Árva J., Fülöp F. (2015). Novel chemo-enzymatic route to a key intermediate for the taxol side-chain through enantioselective *O*-acylation. Unexpected acyl migration. J. Mol. Catal. B.

[59] Yamashita M., Taya N., Nishitani M., Oda K., Kawamoto T., Kimura E., Ishichi Y., Terauchi J., Yamano T. (2015). Preparation of (*S*)-4-(1-(3,4-dichlorophenyl)-2-methoxyethyl)piperidine. Tetrahedron.

[60] Zhou X., Zheng D., Cui B., Han W., Chen Y. (2015). Novozym 435 lipase mediated enantioselective kinetic resolution: A facile method for the synthesis of chiral tetrahydroquinolin-4-ol and tetrahydro1*H*-benzo[*b*]azepin-5-ol derivatives. Tetrahedron.

[61] Villa-Barro A., Gotor V., Brieva R. (2015). Highly selective chemoenzymatic synthesis of enantiopure orthogonally protected *trans*-3-amino-4-hydroxypiperidines. Tetrahedron.

[62] Méndez-Sánchez D., Ríos-Lombardía N., Gotor V., Gotor-Fernández V. (2015). Asymmetric synthesis of azolium-based 1,2,3,4-tetrahydronaphthalen-2-ols through lipase-catalyzed resolutions. Tetrahedron.

[63] Petrenz A., de Maria P.D., Ramanathan A., Hanefeld U., Ansorge-Schumacher M.B., Kara S. (2015). Medium and reaction engineering for the establishment of a chemo-enzymatic dynamic kinetic resolution of *rac*-benzoin in batch and continuous mode. J. Mol. Catal. B.

[64] Hoyos P., Sansottera G., Fernández M., Molinari F., Sinisterra J.V., Alcántara A.R. (2008). Enantioselective monoreduction of different 1,2-diaryl-1,2-diketones catalysed by lyophilised whole cells from *Pichia glucozyma*. Tetrahedron.

[65] Fragnelli M.C., Hoyos P., Romano D., Gandolfi R., Alcántara A.R., Molinari F. (2012). Enantioselective reduction and deracemisation using the non-conventional yeast *Pichia glucozyma* in water-organic solvent biphasic systems: Preparation of (*S*)-1,2-diaryl-2-hydroxyethanones (benzoins). Tetrahedron.

[66] De Miranda A.S., Miranda L.S.M., de Souza R.O.M.A. (2015). Lipases: Valuable catalysts for dynamic kinetic resolutions. Biotechnol. Adv..

[67] Ainge D., Gnad F., Sinclair R., Vaz L.M., Wells A. (2009). Use of Intermediates (*R*)-2,2,4-Trimethyl-l,3-dioxolane-4-yl) methanol (a), 3-Fluoro-4-nitro-phenol (b) and 1-(4-Chloro-benzyl)-piperidin-4-ylamine (c).

[68] Shapiro E., Fishman A., Effenberger R., Maymon A., Schwartz A. (2012). Selective Enzymatic Esterification and Solvolysis of Epimeric Vitamin D Analog and Separation of the Epimers.

[69] Bouzemi N., Grib I., Houiene Z., Aribi-Zouioueche L. (2014). Enantiocomplementary preparation of (*S*)- and (*R*)-arylalkylcarbinols by lipase-catalysed resolution and Mitsunobu inversion: Impact of lipase amount. Catalysts.

[70] López-Iglesias M., Busto E., Gotor V., Gotor-Fernández V. (2015). Chemoenzymatic asymmetric synthesis of 1,4-benzoxazine derivatives: Application in the synthesis of a levofloxacin precursor. J. Org. Chem..

